# QSOX2‐Mediated Disulfide Bond Modification Enhances Tumor Stemness and Chemoresistance by Activating TSC2/mTOR/c‐Myc Feedback Loop in Esophageal Squamous Cell Carcinoma

**DOI:** 10.1002/advs.202500597

**Published:** 2025-05-28

**Authors:** Wo‐Ming Chen, Xiao‐Ping Zhang, Xiao Sun, Hai‐Cheng Liu, Yuan‐Yuan Yan, Xue Wei, Yu Liang, Yue Feng, Zhengjie Chen, Yongxu Jia, Chen Jiang, Qian Yan, Lei Li

**Affiliations:** ^1^ Guangdong Provincial Key Laboratory of Malignant Tumor Epigenetics and Gene Regulation Guangdong‐Hong Kong Joint Laboratory for RNA Medicine Medical Research Center Sun Yat‐sen Memorial Hospital Sun Yat‐Sen University Guangzhou 510120 P. R. China; ^2^ Department of Clinical Laboratory The Sixth Affiliated Hospital Sun Yat‐sen University Guangzhou 510655 P. R. China; ^3^ Breast Tumor Center Sun Yat‐Sen Memorial Hospital Sun Yat‐Sen University Guangzhou 510120 P. R. China; ^4^ Department of Clinical Oncology The First Affiliated Hospital of Zhengzhou University Zhengzhou 451191 P. R. China; ^5^ State Key Laboratory of Metabolic Desregulation & Prevention and Treatment of Esphageal Cancer First Affiliated Hospital of Zhengzhou University Zhengzhou University Zhengzhou 451191 P. R. China; ^6^ State Key Laboratory of Oncology in South China Guangdong Provincial Clinical Research Center for Cancer Sun Yat‐sen University Cancer Center Guangzhou 510060 P. R. China; ^7^ Guangdong Institute of Gastroenterology and Guangdong Provincial Key Laboratory of Colorectal and Pelvic Floor Diseases The Sixth Affiliated Hospital Sun Yat‐sen University Guangzhou 510655 P. R. China

**Keywords:** QSOX2, cancer stem cell, chemoresistance, disulfide bond modification, mTOR signaling

## Abstract

Disulfide bond modification is critical in maintaining protein structure and activity, but its roles in regulating tumor stemness and chemoresistance remain underexplored. Here, Quiescin Sulfhydryl Oxidase 2 (QSOX2) is identified, a protein involved in disulfide bond formation, is highly expressed in esophageal squamous cell carcinoma (ESCC), and is associated with poor patient prognosis. Functional analyses demonstrated that QSOX2 overexpression markedly potentiated tumor stemness and further promoted chemoresistance, proliferation, and metastasis of ESCC cells. Mechanistically, QSOX2 enhances disulfide bond formation in TSC Complex Subunit 2 (TSC2), stabilizing TSC2‐Akt interactions, facilitating phosphorylation of TSC2 at the Ser939 by Akt, and further activating mTOR/4E‐BP1/c‐Myc signaling axis. Intriguingly, cancer‐associated fibroblasts‐secreted IGF‐1 upregulates QSOX2 expression via IGF1R/Akt/mTOR/c‐Myc pathway, establishing a positive feedback loop that sustains ESCC cell stemness. Targeting QSOX2 with Ebselen, in combination with mTOR inhibitor Rapamycin and chemotherapy, effectively downregulates c‐Myc expression and induces tumor dormancy in a mouse xenograft model. Therefore, the findings reveal that QSOX2‐mediated disulfide bond modification enhances tumor stemness by activating mTOR signaling, highlighting a promising therapeutic target in ESCC.

## Graphical abstract

High QSOX2 enhances stemness, drug resistance, and metastasis of ESCC cells. QSOX2‐mediated disulfide bond modification activates mTOR/c‐Myc signaling. CAFs‐secreted IGF‐1 drives mTOR/c‐Myc/QSOX2 positive feedback loop. Combining Ebselen, rapamycin, and cisplatin induces tumor dormancy in mice.

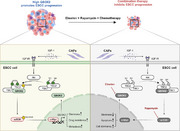

## 1 Introduction

Esophageal cancer is one of the most common and lethal malignancies worldwide, particularly in East Asia. Approximately 90% of esophageal cancer cases are esophageal squamous cell carcinoma (ESCC), with a five‐year survival rate of less than 25%.^[^ ^1^, ^2^ ^]^ Although treatment strategies have diversified in recent years, curative outcomes remain suboptimal. Chemotherapy drug resistance has emerged as a significant barrier, leading to treatment failure and tumor recurrence.^[^ ^3^, ^4^ ^]^ Our previous studies found that cancer stem cells (CSCs) are resistant to chemotherapy and can also maintain a dormant state, contributing to cancer relapse.^[^ ^5^, ^6^ ^]^ Therefore, investigating the molecular mechanisms regulating tumor stemness is crucial for overcoming drug resistance in ESCC.

Protein modification has been shown to be involved in regulating tumor stemness.^[^ ^7^, ^8^, ^9^, ^10^ ^]^ For instance, O‐GlcNAc transferase modifies eIF4E via O‐GlcNAcylation to promote the stem‐like characteristics of liver CSCs.^[^ ^9^ ^]^ Histone Deacetylase 1 (HDAC1) influences Wnt and Notch signaling pathways in colorectal CSCs via deacetylation, thereby supporting tumor stemness.^[^ ^10^ ^]^ Recently, disulfide bond formation, an important post‐translational modification of proteins, has been increasingly recognized as playing a critical role in protein folding and functional maintenance.^[^ ^11^ ^]^ However, the specific function of disulfide bond modification in tumor stemness remains underexplored. Using public sequencing data to analyze the expression levels of genes associated with disulfide bond formation in ESCC and normal esophageal tissues, we found that Quiescin Sulfhydryl Oxidase 2 (QSOX2) was highly expressed in ESCC.

QSOX is a disulfide catalyst that oxidizes thiols and contributes to disulfide bond formation during protein folding.^[^ ^12^ ^]^ Recent studies have found that overexpressed QSOX2 is associated with poor survival in patients with non‐small cell lung cancer, colorectal cancer, or prostate cancer.^[^ ^13^, ^14^, ^15^ ^]^ QSOX2 silencing inhibits lung cancer cell proliferation, induces cell apoptosis, and reduces the expression of genes related to cell division (CENPF and NUSAP1) as well as Wnt pathway activators, including PRRX2 and Nuc‐β‐catenin.^[^ ^13^ ^]^ Moreover, QSOX2 exacerbates the progression of triple‐negative breast cancer by increasing the stability of integrin β‐1.^[^ ^16^ ^]^ In addition, QSOX2 is involved in sperm production and maturation through gain or loss of function in animal models.^[^ ^17^ ^]^ These evidences suggest that QSOX2 may be involved in regulating tumor stemness. However, the correlation between QSOX2 and CSCs has not yet been reported.

In the present study, we found that overexpression of QSOX2 enhanced tumor stemness and chemoresistance, contributing to poor prognosis in ESCC. Mechanistically, QSOX2 enhances disulfide bond and phosphorylation modifications in TSC Complex Subunit 2 (TSC2), activating mTOR/4E‐BP1/c‐Myc signaling axis. Furthermore, cancer‐associated fibroblasts (CAFs)‐activated IGF1R/Akt/mTOR/c‐Myc pathway transcriptionally upregulates QSOX2 expression, establishing a QSOX2/mTOR feedback loop that sustains ESCC cell stemness. Combining QSOX2 inhibitor Ebselen, mTOR inhibitor Rapamycin, and chemotherapy reduces ESCC stemness and induces tumor dormancy. These findings offer a promising therapeutic approach for ESCC patients with high QSOX2 expression.

## 2 Results

### 2.1 High QSOX2 is Associated with Poor Prognosis in ESCC Patients

**Figure 1:**
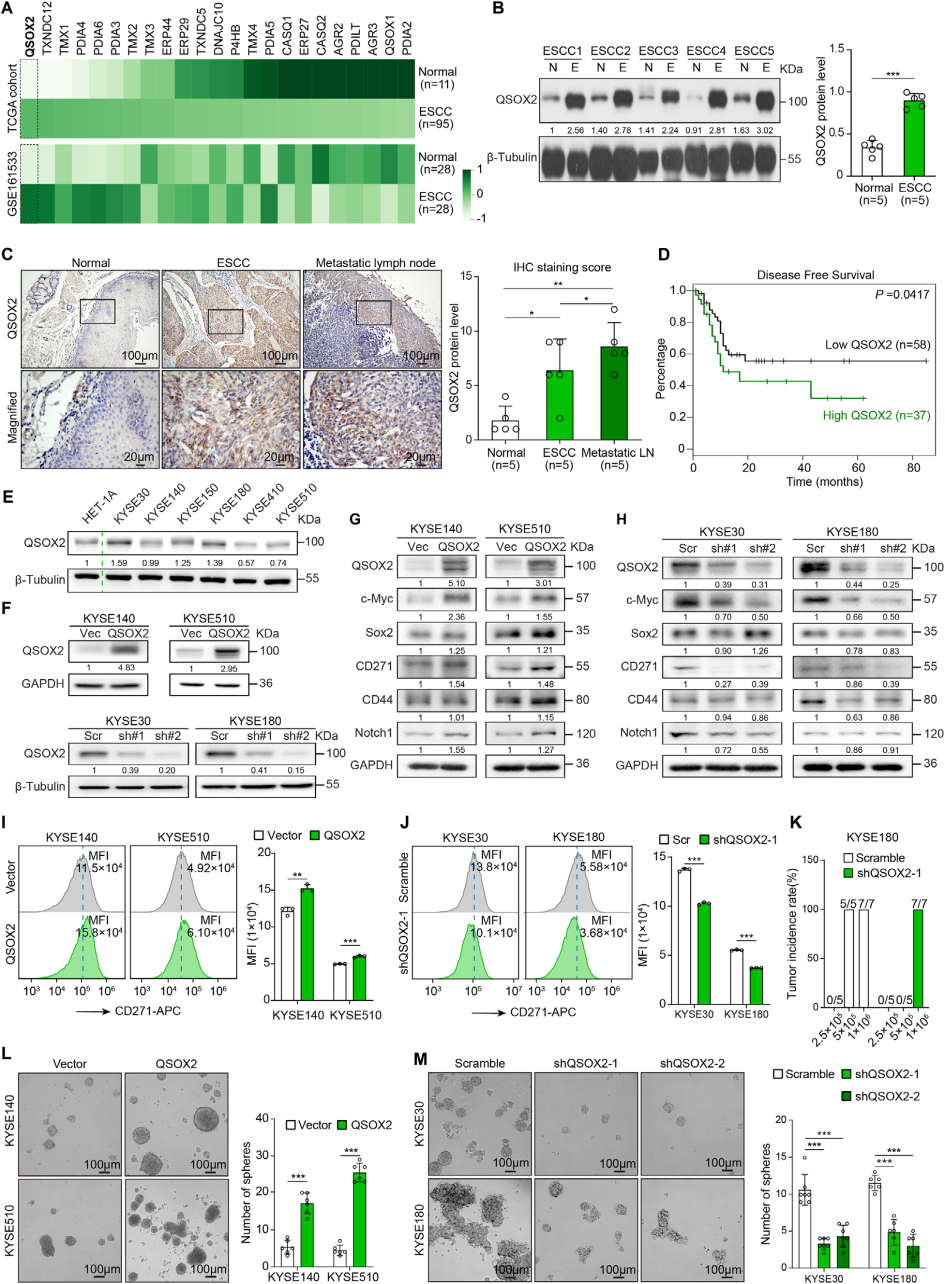
High QSOX2 enhances stemness, resulting in poor prognosis in ESCC. A) Heat maps showed that *QSOX2* was significantly upregulated in ESCC tumors compared to normal esophageal tissues. B) Western blot was used to assess the protein level of QSOX2 in five pairs of ESCC tumors (E) and adjacent normal tissues (N). β‐Tubulin was used as a control. C) IHC staining was employed to analyze the protein level of QSOX2 in adjacent normal tissues, ESCC tumors, and metastatic lymph nodes. D) Kaplan–Meier survival curves demonstrated that a high level of QSOX2 was associated with poor prognosis in ESCC patients. E) Western blot was used to assess the protein level of QSOX2 in immortalized esophageal epithelial cell HET‐1A and six ESCC cell lines. F) Western blot was performed to confirm the overexpression or silence of QSOX2 in ESCC cells. G, H) Western blot was used to detect the expression of stemness markers after QSOX2 overexpression or knockdown. I, J) Flow Cytometry was conducted to measure the level of CD271 in ESCC cells after QSOX2 overexpression or silence. MFI, Mean Fluorescence Intensity. K) Tumor incidence was evaluated in BALB/c‐nude mice one month after injection of ESCC cells with a gradient cell count. L, M) Sphere formation assay was performed to assess the stemness of ESCC cells following QSOX2 overexpression or knockdown. In all panels, data are presented as the mean ± SD; In panels B and C, data were analyzed using paired two‐tailed Student's t‐test with Welch's correction; In panels I‐M, data were analyzed using unpaired two‐tailed Student's t‐test with Welch's correction; *p < 0.05, **p < 0.01, and ***p < 0.001.

To identify key molecules mediating disulfide bond modification in ESCC, we analyzed the mRNA levels of 23 enzymes involved in disulfide bond formation using The Cancer Genome Atlas (TCGA) cohort and three Gene Expression Omnibus (GEO) datasets.^[^ ^18^ ^]^ Results showed that *QSOX2* expression was higher in ESCC compared to normal esophageal tissues in all these databases (**Figure** **1**A; Figure S1A,B, Supporting Information). Disease Ontology analysis also indicated a significant association between *QSOX2* and cancer (Figure S1C, Supporting Information). Western blot (Figure 1B) and immunohistochemistry (IHC) staining (Figure 1C) confirmed the increased QSOX2 protein levels in clinical ESCC samples compared to paired non‐tumor esophageal tissues. Data from the UALCAN database (https://ualcan.path.uab.edu/) also demonstrated that QSOX2 protein expression was elevated in various tumors relative to normal tissues (Figure S1D, Supporting Information). Furthermore, QSOX2 expression was higher in metastatic lymph nodes than in the primary tumor (Figure 1C; Figure S1E, Supporting Information). Clinical data from the TCGA database further showed that *QSOX2* mRNA level was higher in poorly differentiated tumors (Figure S1F, Supporting Information). Furthermore, score assessments using transcriptome or single‐cell data showed that ESCC tissues and cells with high QSOX2 expression had higher stemness scores, as well as higher metastasis and proliferation scores (Figure S2A,B, Supporting Information), implying that ESCC cells with high QSOX2 expression have higher stemness. Kaplan‐Meier survival curve showed that ESCC patients with high *QSOX2* expression had shorter progression‐free survival compared to those with low *QSOX2* expression (Figure 1D). However, univariate and multivariate analyses showed that QSOX2 expression was not an independent risk factor for ESCC patients (Table S3, Supporting Information). Therefore, increased QSOX2 may promote ESCC progression by regulating tumor stemness.

### 2.2 QSOX2 Enhances Stemness and Drug Resistance of ESCC Cells

To explore the correlation between QSOX2 and tumor stemness, we overexpressed or silenced QSOX2 expression in ESCC cells. We collected proteins from six ESCC cell lines (KYSE30, KYSE140, KYSE150, KYSE180, KYSE410, and KYSE510) and the esophageal epithelial cell line HET‐1A. Western blot analysis showed that QSOX2 protein levels were higher in KYSE30, KYSE150, and KYSE180 cells, but lower or similar in KYSE140, KYSE410, and KYSE510 cells compared to HET‐1A cells (Figure 1E). Subsequently, we overexpressed QSOX2 in KYSE140 and KYSE510 cells with low QSOX2 expression and silenced QSOX2 in KYSE30 and KYSE180 cells with high QSOX2 expression using a lentiviral transfection system (Figure 1F). Next, western blot demonstrated that the expression of stemness‐associated transcription factors (c‐Myc and Sox2) and esophageal CSC markers (CD271, CD44, and Notch1) was increased in ESCC cells with QSOX2 overexpression and decreased in ESCC cells after QSOX2 knockdown (Figure 1G,H). Flow cytometry and IF staining also revealed that QSOX2 overexpression upregulated the level of CD271 (Figure 1I; Figure S2C, Supporting Information), while QSOX2 silencing reduced CD271 expression (Figure 1J; Figure S2D, Supporting Information). Limited dilution experiment in immunocompromised and immunocompetent mice indicated that ESCC cells with low expression of QSOX2 were unable to form xenograft tumors when transplanted in fewer cells (Figure 1K; Figure S2E,F, Supporting Information). Furthermore, sphere formation assay showed that QSOX2 overexpression significantly increased the frequency of sphere formation (Figure 1L), while QSOX2 silencing decreased this frequency (Figure 1M). Collectively, high QSOX2 enhances tumor stemness in ESCC.

**Figure 2:**
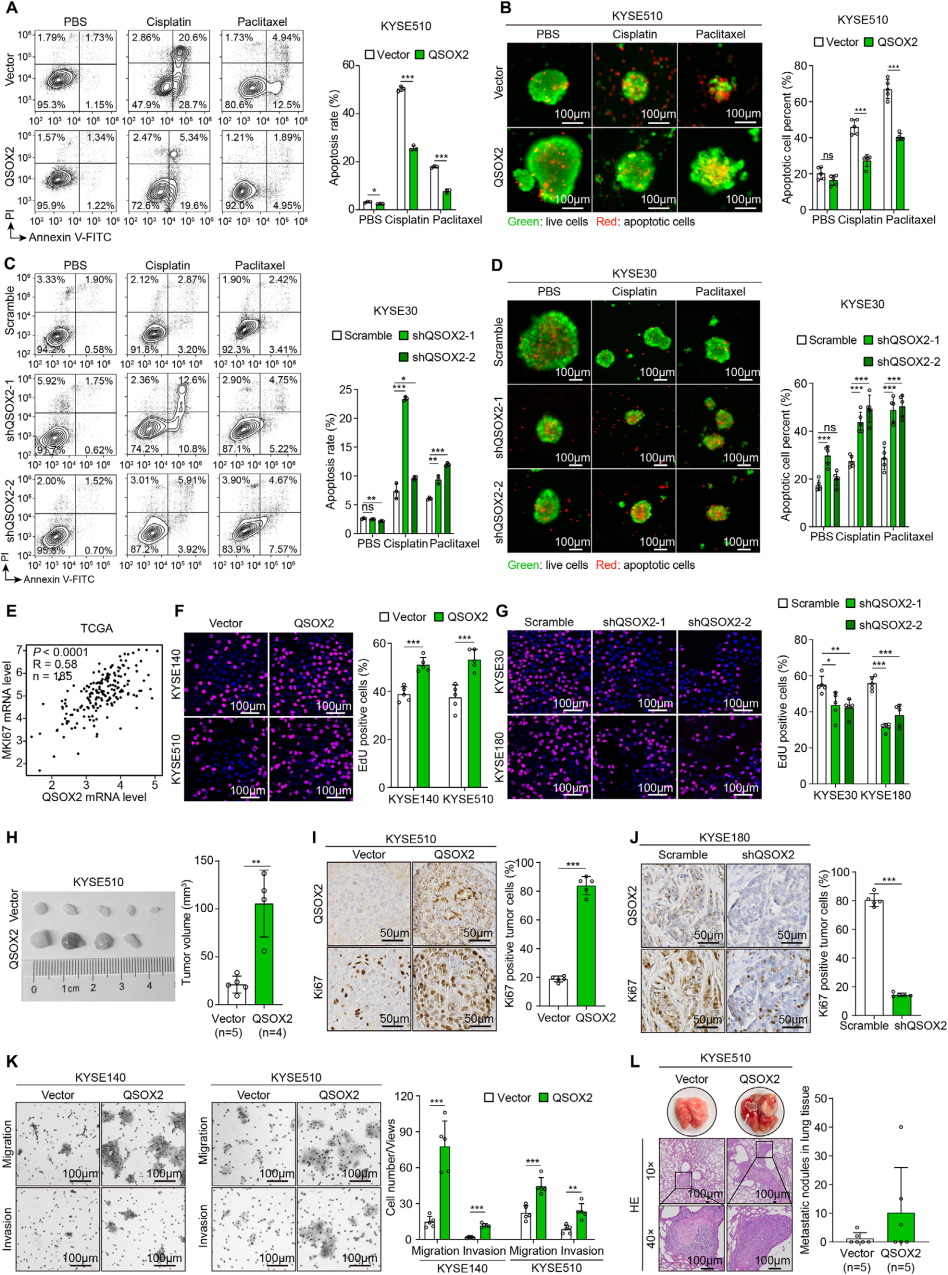
QSOX2 promotes chemoresistance, proliferation, and metastasis in ESCC cells. A) Cell apoptosis assays evaluated the chemosensitivity of ESCC cells with QSOX2 overexpression. B) Calcein AM/PI double staining was performed to test the apoptotic cell rate of QSOX2 overexpressed ESCC cells under treatments with Cisplatin or Paclitaxel. C) Cell apoptosis assays showed the increased chemosensitivity of ESCC cells with QSOX2 silence. D) Calcein AM/PI double staining was used to analyze the apoptotic cell rate of QSOX2‐silenced ESCC cells treated with Cisplatin or Paclitaxel. E) Co‐expression analysis between *QSOX2* and *MKI67* in ESCA using the TCGA cohort. F, G) EdU staining was performed to test the cell proliferation rate of ESCC cells with QSOX2 overexpression or silence. H) Xenograft tumor experiment was performed using KYSE510‐Vector and KYSE510‐QSOX2 cells, and tumor volume was calculated. I, J) IHC staining with antibodies against QSOX2 and Ki67 was performed on xenograft tumors derived from QSOX2‐overexpressed KYSE510 cells (I) or QSOX2‐silenced KYSE180 cells (J). K) Transwell assay showed the enhanced cell migration and invasion abilities of ESCC cells with QSOX2 overexpression. L) A lung metastasis experiment was performed to analyze the metastatic ability of KYSE510‐Vector and KYSE510‐QSOX2. In I and J panels, data are presented as the mean ± SEM; other panels, data are presented as the mean ± SD; unpaired two‐tailed Student's t‐test with Welch's correction; *p < 0.05, **p < 0.01, and ***p < 0.001. ns, no significant difference.

Since tumor stemness plays a pivotal role in regulating chemosensitivity, we further investigated whether QSOX2 contributes to chemotherapy drug resistance. First, GEO datasets analyses revealed that QSOX2 expression was significantly elevated in Cisplatin‐resistant ESCC patients and cell lines compared to their Cisplatin‐sensitive counterparts (Figure S3A, Supporting Information). Consistent with this, Cisplatin IC50 assays showed that QSOX2‐overexpressing ESCC cells tolerated higher drug concentrations than their corresponding control cells (Figure S3B, Supporting Information). Conversely, QSOX2‐silenced KYSE30 cells displayed a 50% growth inhibition rate at a lower drug concentration (Figure S3C, Supporting Information). Furthermore, cell apoptosis assay and Calcein AM/PI double staining showed that KYSE510 and KYSE140 cells with QSOX2 overexpression displayed a reduced apoptosis rate under treatments of Cisplatin or Paclitaxel (**Figure** **2**A,B, Figure S3D,E, Supporting Information). In contrast, KYSE30 and KYSE180 cells with QSOX2 silence exhibited increased sensitivity to Cisplatin or Paclitaxel treatments (Figure 2C,D, Figure S3F,G, Supporting Information). In summary, these findings indicate that QSOX2 enhances tumor stemness and promotes chemotherapeutic drug resistance in ESCC cells.

### 2.3 High QSOX2 Drives ESCC Cell Proliferation and Metastasis

In addition, correlation analysis of gene expression using TCGA data revealed a significant positive association between *QSOX2* and the cell proliferation markers *MKI67* and *PCNA* in the esophageal carcinoma (ESCA) cohort (Figure 2E; Figure S4A, Supporting Information). Single‐sample gene set enrichment analysis (ssGSEA) based on TCGA data further indicated that QSOX2 was involved in tumor cell proliferation and DNA replication (Figure S4B, Supporting Information). These bioinformatics analyses strongly support the critical role of QSOX2 in regulating cell proliferation. EdU incorporation assays demonstrated that increased QSOX2 expression significantly enhanced the proliferation of KYSE140 and KYSE510 cells (Figure 2F). Conversely, silencing QSOX2 expression markedly suppressed the proliferation of KYSE30 and KYSE180 cells (Figure 2G). Results of subcutaneous tumor transplantation in immunocompromised and immunocompetent mice showed that tumors derived from QSOX2‐overexpressing KYSE510 or mEC2 cells exhibited larger volumes and heavier weights (Figure 2H; Figure S4C,D, Supporting Information). In contrast, knockdown of QSOX2 in KYSE180 cells suppressed tumor growth (Figure S4E, Supporting Information). IHC staining further confirmed that tumors derived from QSOX2‐overexpressing KYSE510 cells displayed elevated Ki67 levels compared to the control tumors (Figure 2I), whereas tumors derived from QSOX2‐silenced KYSE180 cells showed reduced Ki67 levels (Figure 2J). Therefore, high QSOX2 expression promotes ESCC cell proliferation.

We further investigated the effect of QSOX2 on the metastatic potential of ESCC cells. In vitro transwell assays showed that increased QSOX2 expression significantly enhanced the migratory and invasive abilities of KYSE140 and KYSE510 cells (Figure 2K). Conversely, silencing QSOX2 expression markedly inhibited the migratory and invasive capacities of KYSE30 and KYSE180 cells (Figure S4F, Supporting Information). The lung metastasis model revealed that KYSE510 cells with QSOX2 overexpression formed more metastatic nodules compared to the control group (Figure 2L). These data indicate that QSOX2 promotes ESCC cell metastasis.

### 2.4 QSOX2 Enhances Stemness by Upregulating c‐Myc

**Figure 3:**
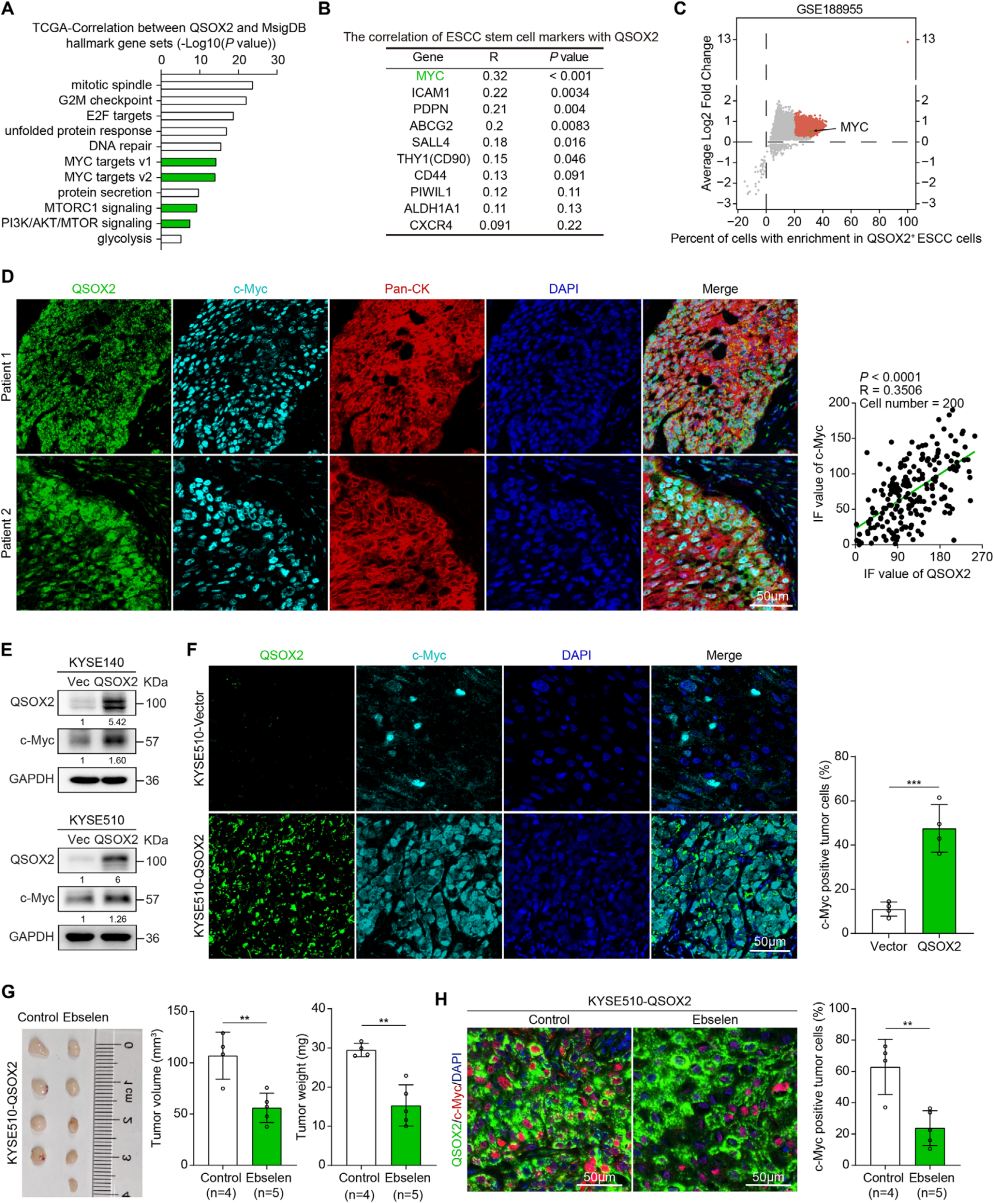
QSOX2 enhances tumor stemness by upregulating c‐Myc. A) Correlation between *QSOX2* and MsigDB hallmark gene sets in the TCGA cohort. B) Co‐expression analysis between *QSOX2* and cell stemness markers in ESCA using the TCGA cohort. C) Volcano plots illustrate the differentially expressed genes between QSOX2^+^ and QSOX2^−^ ESCC cell populations using the GEO dataset (GSE188955). D) Quantification of c‐Myc and QSOX2 staining intensity in ESCC patient tissues (*n* = 200 cells) using ImageJ software, followed by linear regression analysis. E) Western blot analysis of c‐Myc expression after QSOX2 overexpression in ESCC cells. F) Percentage of c‐Myc positive tumor cells in xenograft tumors derived from QSOX2‐ or vector‐transfected KYSE510 cells was analyzed by multiplex IF staining. G) Xenograft tumors derived from KYSE510‐QSOX2 cells were treated with Ebselen (5 mg kg^−1^, i.p.) every three days for three times 12 days after cell injection, and tumor volume and weight were measured. H) Double IF staining with antibodies against QSOX2 and c‐Myc was performed on KYSE510‐QSOX2‐derived xenograft tumors after treatment with Ebselen. In F and H panels, data are presented as the mean ± SEM; in panel G, data are presented as the mean ± SD; unpaired two‐tailed Student's t‐test with Welch's correction; **p < 0.01, and ***p < 0.001.

To explore the mechanism by which QSOX2 regulates tumor stemness, we analyzed its correlation with MsigDB hallmark gene sets using the TCGA database. The analysis revealed that QSOX2 expression was significantly associated with *MYC* target gene sets, including *PLK1*, *CBX3*, and *HDAC2* (**Figure** **3**A; Figure S5A, Supporting Information). Further correlation analysis of stemness markers in ESCC showed a significant positive association between *QSOX2* and *MYC* (Figure 3B; Figure S5B, Supporting Information). In addition, we analyzed the scRNA‐Seq dataset (GSE188955) and found that QSOX2^+^ ESCC cells had higher *MYC* mRNA levels than QSOX2^−^ ESCC cells (Figure 3C). IF staining also demonstrated a significant positive correlation between QSOX2 and c‐Myc expression at the protein level in clinical ESCC tissues (Figure 3D). Western blot analysis revealed that c‐Myc expression was significantly increased following QSOX2 overexpression in KYSE140 and KYSE510 cells (Figure 3E). Double IF staining analysis of xenograft tumors further confirmed that QSOX2 overexpression increased c‐Myc expression in xenograft tumors (Figure 3F). In addition, subcutaneous xenograft tumors derived from QSOX2‐overexpressing KYSE510 cells were treated with QSOX2 inhibitor Ebselen (5 mg kg^−1^, i.p.). Compared to the control group, the Ebselen treatment reduced tumor volume and weight (Figure 3G). Importantly, the level of c‐Myc^+^ ESCC cells was decreased in Ebselen‐treated tumors (Figure 3H). Overall, these findings indicate that QSOX2 enhances tumor stemness by upregulating c‐Myc expression.

### 2.5 QSOX2 Activates the mTOR/c‐Myc Signaling by Increasing p‐TSC2^Ser939^

**Figure 4:**
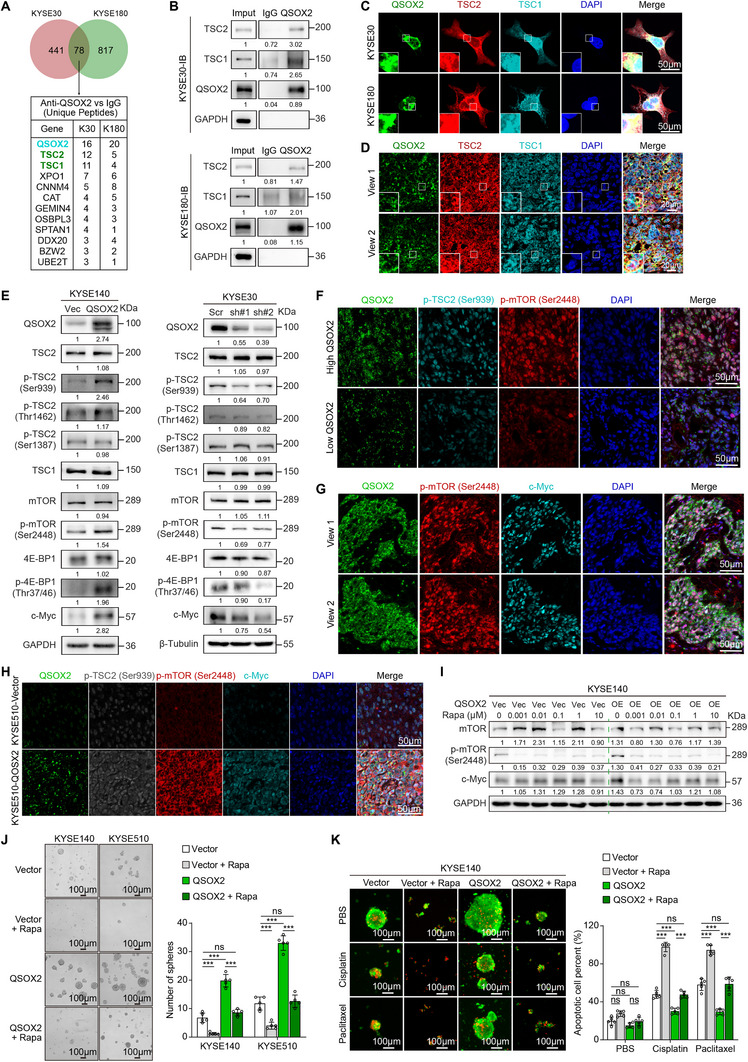
QSOX2 activates the mTOR/c‐Myc signaling by promoting the phosphorylation of TSC2 at Ser939 site. A) LC‐MS/MS analysis of QSOX2 binding proteins. B) Protein IP assay was performed with QSOX2 antibody on KYSE30 and KYSE180 cells. C) Multiplex IF staining showed co‐localization of QSOX2, TSC1, and TSC2 in KYSE30 and KYSE180 cells. D) Multiplex IF staining showed co‐localization of QSOX2, TSC1, and TSC2 in ESCC patient tissues. E) Western blot was used to analyze the activation or inactivation of TSC2/mTOR/4E‐BP1/c‐Myc signaling after QSOX2 overexpression or silence in ESCC cells. F) The levels of QSOX2, phosphorylated TSC2 (p‐TSC2^Ser939^), and phosphorylated mTOR (p‐mTOR^Ser2448^) in ESCC patient tissues with high or low QSOX2 expression were analyzed by multiplex IF staining. G) Multiplex IF staining showed the co‐expression of QSOX2, p‐mTOR^Ser2448^ and c‐Myc in ESCC patient tissues. H) Multiplex IF staining showed the levels of QSOX2, p‐TSC2^Ser939^, p‐mTOR^Ser2448^ and c‐Myc in xenograft tumors. I) Western blot was performed to test the levels of p‐mTOR^Ser2448^ and c‐Myc in KYSE140‐Vector and KYSE140‐QSOX2 cells treated with different concentrations of Rapamycin (24 h). J) Sphere formation assay to evaluate stemness in QSOX2‐overexpressing ESCC cells treated with Rapamycin (10 µM, 24 h). K) Calcein AM/PI double staining was performed to analyze the effect of Rapamycin on the apoptotic cell rate of KYSE140‐Vector and KYSE140‐QSOX2 cells treated with Cisplatin or Paclitaxel. In J and K panels, data are presented as the mean ± SD; unpaired two‐tailed Student's t‐test with Welch's correction; ***p < 0.001. ns, no significant difference.

To elucidate the molecular mechanism by which QSOX2 upregulates c‐Myc expression, we performed a protein IP assay followed by mass spectrometry to identify QSOX2‐interacting proteins in ESCC cells. Intersecting the potential QSOX2‐binding proteins identified in both KYSE30 and KYSE180 cells yielded 78 common proteins (Table S4, Supporting Information). Notably, TSC2 and TSC1 were among the most frequently identified proteins, excluding QSOX2 itself (**Figure** **4**A; Figure S5C, Supporting Information). IP assays confirmed the interaction between QSOX2 and TSC1/TSC2 (Figure 4B). Multiplex IF staining of ESCC cells and clinical tissues further validated the spatial co‐localization of QSOX2 with TSC1/TSC2 (Figure 4C,D).

TSC2, a key inhibitor of mTORC1, suppresses cell growth and metabolism by negatively regulating mTOR/4EBP1/c‐Myc axis.^[^ ^19^, ^20^ ^]^ Phosphorylation of TSC2 at Ser939 is primarily mediated by Akt, which attenuates TSC2‐mediated inhibition of mTORC1.^[^ ^21^ ^]^ While TSC1 primarily functions to stabilize TSC2, with its absence leading to TSC2 degradation,^[^ ^22^ ^]^ we focused our subsequent studies on TSC2 as it directly modulates the mTOR pathway. Western blot analysis showed that QSOX2 overexpression significantly increased the phosphorylation levels of TSC2^Ser939^, mTOR^Ser2448^, 4E‐BP1^Thr37/46^ and expression level of c‐Myc in ESCC cells, while the levels of p‐TSC2^Thr1462^ and p‐TSC2^Ser1387^ remained unchanged (Figure 4E; Figure S5D, Supporting Information). Conversely, knockdown of QSOX2 in KYSE30 and KYSE180 cells decreased the expression of these phosphorylated proteins (Figure 4E; Figure S5D, Supporting Information). Multiplex IF staining revealed that the levels of p‐TSC2^Ser939^ and p‐mTOR^Ser2448^ were elevated in ESCC patient tissues with high QSOX2 expression (Figure 4F). Moreover, p‐mTOR^Ser2448^ level was positively correlated with c‐Myc expression in ESCC patient tissues (Figure 4G). Importantly, p‐TSC2^Ser939^/p‐mTOR^Ser2448^/c‐Myc signaling pathway was activated in subcutaneous xenograft tumors with QSOX2 overexpression (Figure 4H). Furthermore, treatment of QSOX2‐overexpressing KYSE140 and KYSE510 cells with Rapamycin, an mTOR inhibitor, reversed QSOX2‐induced mTOR activation (Figure 4I; Figure S5E, Supporting Information). Sphere formation and Calcein AM/PI double staining assays further demonstrated that Rapamycin treatment mitigated tumor stemness and chemotherapy drug resistance induced by QSOX2 overexpression (Figure 4J,K). Thus, QSOX2 activates the mTOR/4E‐BP1/c‐Myc pathway by promoting p‐TSC2^Ser939^.

### 2.6 QSOX2 Promotes Disulfide Bond Modification Facilitating Akt‐Mediated p‐TSC2^Ser939^

**Figure 5:**
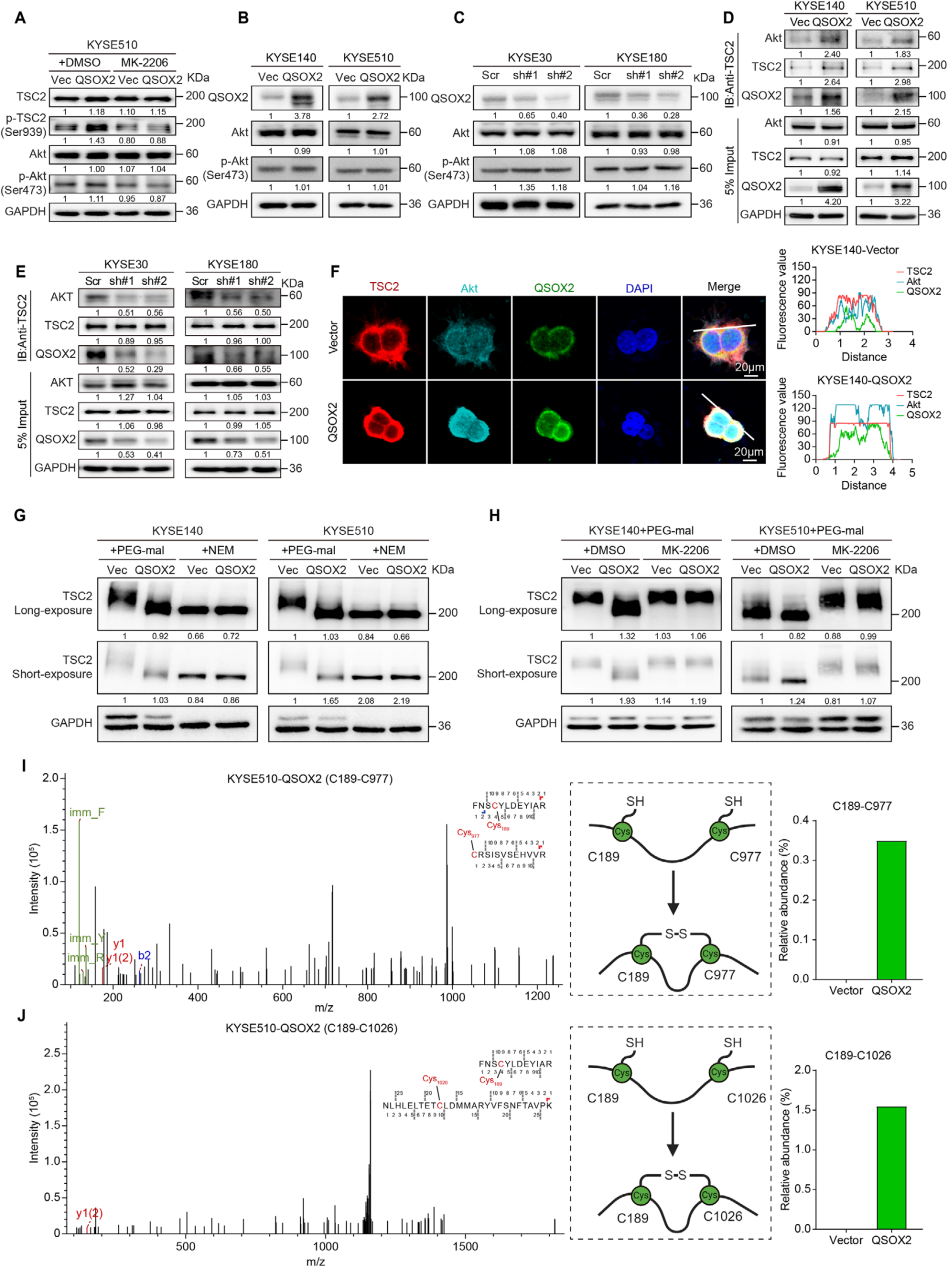
QSOX2 promotes disulfide bond formation and phosphorylation of TSC2 by binding Akt. A) Western blot analysis confirming the activation of p‐TSC2^Ser939^ after QSOX2 overexpression and the inhibition of p‐TSC2^Ser939^ by Akt inhibitor MK‐2206 treatment (5 µM, 24 h). B, C) Western blot analysis showing the effect of QSOX2 on the protein levels of total Akt and p‐Akt^Ser473^ in the indicated ESCC cells. D) Protein IP assay was performed with TSC2 antibody on ESCC cells with or without QSOX2 overexpression. E) Protein IP assay was performed with TSC2 antibody on ESCC cells with or without QSOX2 silence. F) Multiplex IF demonstrated co‐localization of TSC2, Akt, and QSOX2 in KYSE140‐Vector and KYSE140‐QSOX2 cells. G) Western blot showed the protein band location of TSC2 from Vector‐ or QSOX2‐transfected ESCC cells. Protein lysates were treated with polyethylene glycol maleimide (PEG‐mal) or N‐ethylmaleimide (NEM) as indicated above each blot. PEG‐mal can alkylate free cysteine residues that have not formed disulfide bonds, thereby increasing the molecular weight of the protein. H) ESCC cells were treated with Akt inhibitor MK‐2206 (5 µM, 24 h), and protein lysates were treated with PEG‐mal as indicated above each blot. Western blot analyzed the protein band location of TSC2 from Vector‐ or QSOX2‐transfected ESCC cells. I, J) Mass spectrometry analysis coupled with quantitative bar chart visualization revealed alterations in the disulfide bond formation between C189 and C977/C1026 residues of TSC2 protein in KYSE510‐Vector versus KYSE510‐QSOX2 cells.

A previous study has established that Akt mediates TSC2 phosphorylation at Ser939.^[^ ^21^ ^]^ Therefore, we treated K510‐Vector and K510‐QSOX2 cells with an Akt inhibitor MK‐2206. Results of western blot showed that MK‐2206 treatment reduced the level of p‐TSC2^Ser939^, suggesting that QSOX2 overexpression leads to TSC2 phosphorylation at Ser939 via Akt (**Figure** **5**A). Notably, altering QSOX2 expression levels did not significantly affect total Akt and p‐Akt^Ser473^ levels (Figure 5B,C). However, IP assay revealed that QSOX2 overexpression strengthened the interaction between TSC2 and Akt in KYSE140 and KYSE510 cells (Figure 5D), and QSOX2 knockdown weakened the interaction between TSC2 and Akt in KYSE30 and KYSE180 cells (Figure 5E). Colocalization analysis confirmed increased spatial proximity between TSC2 and Akt in the presence of elevated QSOX2 in KYSE140 cells (Figure 5F). These findings suggest that QSOX2 facilitates TSC2 phosphorylation at Ser939 by enhancing the binding between TSC2 and Akt, rather than by directly modulating Akt activity.

QSOX2 has been reported to be involved in disulfide bond formation,^[^ ^23^, ^24^ ^]^ so we analyzed whether QSOX2 is modified by regulating disulfide bonds in TSC2. Polyethylene glycol maleimide (PEG‐mal) is commonly used to modify proteins by alkylating free cysteine residues that are not engaged in disulfide bonds.^[^ ^25^ ^]^ Adding PEG‐mal to the protein lysates of KYSE140 and KYSE510 cells revealed that the molecular weight of TSC2 in control cells was higher than that in QSOX2‐overexpressed ESCC cells, indicating that there were fewer free cysteine residues and more disulfide bonds formed after QSOX2 overexpression (Figure 5G). As a control, the molecular weight of TSC2 did not change in vector‐ or QSOX2‐transfected ESCC cells after N‐ethylmaleimide (NEM) treatment (Figure 5G). Intriguingly, treatment with Akt inhibitor MK‐2206 led to an increase in the molecular weight of TSC2 in the protein lysates from ESCC cells supplemented with PEG‐mal, suggesting that both QSOX2 and Akt are involved in the disulfide bond formation of TSC2 (Figure 5H). Moreover, molecular docking experiments revealed that Akt and QSOX2 bind to the region spanning amino acids 151–310 and 162–398 of TSC2, respectively. Their interacting protein region consists of one cysteine residue Cys189 that may be catalyzed by QSOX2 to form a disulfide bond (Figure S6A, Supporting Information). KYSE510‐Vector and KYSE510‐QSOX2 were used for mass spectrometry analysis of disulfide bonds of TSC2. The results showed that TSC2 protein increased two disulfide binding sites, Cys189‐Cys977 and Cys189‐Cys1026, after overexpression of QSOX2 (Figure 5I,J). Therefore, QSOX2 promotes phosphorylation and disulfide bond modifications of TSC2 by interacting Akt.

### 2.7 CAFs‐Secreted IGF‐1 Upregulates QSOX2 via Akt/mTOR/c‐Myc Signaling Axis

**Figure 6:**
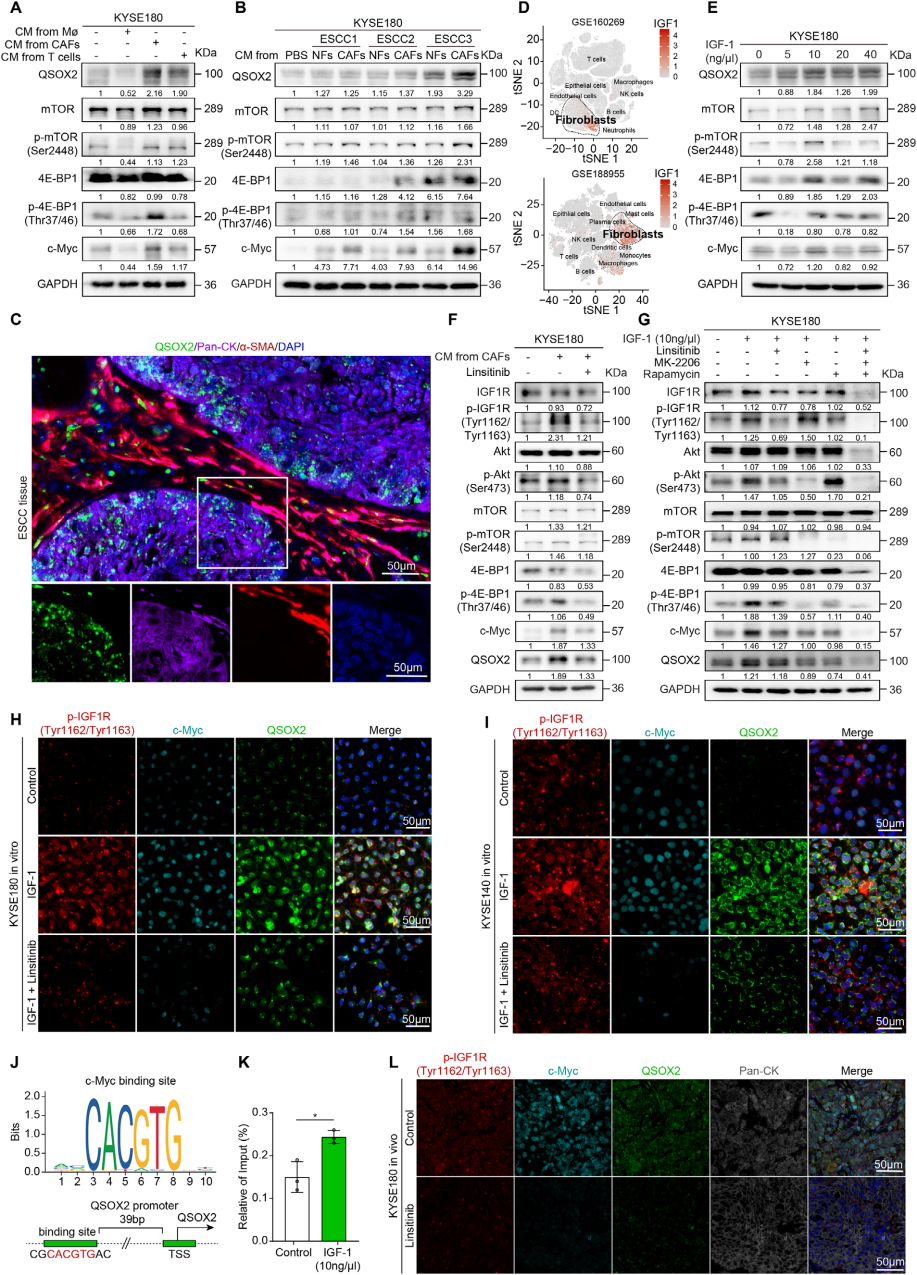
CAFs‐secreted IGF‐1 upregulates the Akt/mTOR/c‐Myc/QSOX2 signaling. A) CAFs‐conditioned media upregulated the levels of QSOX2, p‐mTOR, p‐4E‐BP1, and c‐Myc in KYSE180 cells. B) Western blot was used to test the levels of QSOX2, p‐mTOR, p‐4E‐BP1, and c‐Myc in KYSE180 cells treated with fibroblasts‐conditioned media. NFs, normal fibroblasts; CAFs, cancer‐associated fibroblasts. C) Multiplex IF staining confirmed that CAFs (α‐SMA positive) were adjacent to QSOX2‐expressed ESCC cells. D) Single‐cell RNA sequencing from GEO datasets (GSE160269 and GSE188955) was performed to identify the main cell populations expressing IGF‐1. E) The levels of QSOX2, p‐mTOR, p‐4E‐BP1, and c‐Myc in KYSE180 cells treated with different concentrations of IGF‐1 (24 h) were analyzed by western blot. F) Western blot confirmed the activation of IGF1R/Akt/mTOR/c‐Myc/QSOX2 signaling in KYSE180 cells by CAFs‐conditioned media, and this stimulation was inhibited by IGF1R inhibitor Linsitinib (5 µM, 24 h). G) Western blot showed the activation of IGF1R/AKT/mTOR/c‐Myc/QSOX2 signaling by IGF‐1 treatment (10 ng µL^−1^, 24 h), and this stimulation was inhibited by Linsitinib (5 µM), MK‐2206 (5 µM), or Rapamycin (10 µM). H, I) Multiplex IF staining confirmed the activation of IGF1R/c‐Myc/QSOX2 signaling by IGF‐1 treatment (10 ng µL^−1^, 24 h), and this stimulation was inhibited by Linsitinib (5 µM) in KYSE180 (H) and KYSE140 (I) cells. J) The transcriptional binding site of c‐Myc in the *QSOX2* gene promoter. K) ChIP‐qPCR analysis showing that IGF‐1 (10 ng µL^−1^, 24 h) stimulation promoted the binding of c‐Myc to the promoter of *QSOX2* gene. L) Multiplex IF staining was performed to analyze the levels of p‐IGF1R, c‐Myc and QSOX2 on KYSE180‐derived xenograft tumors treated with or without Linstinib (25 mg kg^−1^, i.g.). In panel K, data are presented as the mean ± SD; unpaired two‐tailed Student's t‐test with Welch's correction; *p < 0.05.

QSOX2 is often found to be highly expressed at the edge of tumor nests, suggesting that the microenvironment may regulate QSOX2 expression (Figure S7A, Supporting Information). Hence, KYSE180 cells were treated with conditioned media from macrophages, CAFs, and T cells for 24 h, respectively. Results of western blot showed that conditioned media derived from CAFs, but not normal fibroblasts, markedly increased QSOX2 expression and activated the mTOR/4E‐BP1/c‐Myc signaling pathway (**Figure** **6**A,B). Multiplex IF staining revealed that ESCC cells with high QSOX2 expression were surrounded by abundant CAFs (Figure 6C). Moreover, the expression level of QSOX2 was positively correlated with the infiltration level of CAFs in the TCGA database (Figure S7B, Supporting Information). IGF‐1 is mainly secreted by CAFs (Figure 6D) and has been reported to activate Akt/mTOR signaling to promote tumor progression.^[^ ^26^ ^]^ To confirm whether IGF‐1 upregulates QSOX2 expression, we treated KYSE180 cells with graded concentrations of IGF‐1 recombinant protein for 24 h. Results of western blot showed that IGF‐1 treatment upregulated QSOX2 expression and activated the mTOR pathway (Figure 6E). Importantly, IGF1R inhibitor Linstinib, Akt inhibitor MK‐2206, and mTOR inhibitor Rapamycin either alone or in combination attenuated the activation of IGF1R/Akt/mTOR/4E‐BP1/c‐Myc signaling axis and the upregulation of QSOX2 in ESCC cells induced by CAFs‐derived conditioned media or IGF‐1 recombinant protein (Figure 6F‐I; Figure S7C, Supporting Information). We further performed CellChat analysis on CAFs, QSOX2^+^ or QSOX2^−^ ESCC cells using scRNA‐Seq datasets. The results revealed that, in addition to the IGF‐1/IGFR axis, CAFs may interact QSOX2^+^ ESCC cells by secreting factors such as HBEGF, TIMP1 or B2M (Figure S7D,E, Supporting Information). Previous study has shown that HBEGF promotes myocardial interstitial fibrosis via the Akt/mTOR pathway,^[^ ^27^ ^]^ suggesting a potential cooperative role with IGF‐1 in modulating QSOX2 expression in tumor cells through Akt/mTOR signaling.

Next, analysis of transcription factor binding sites in the *QSOX2* promoter revealed a c‐Myc binding site upstream of the transcription start site (Figure 6J). ChIP‐qPCR was conducted to investigate the interaction between c‐Myc and the potential binding site in KYSE180 cells treated with or without IGF‐1 (10 ng µL^−1^). Results showed that c‐Myc transcriptionally upregulates QSOX2, and IGF‐1 stimulation enhanced c‐Myc binding to the QSOX2 promoter (Figure 6K). Analysis of GEO dataset (GSE22139) further revealed that QSOX2 expression was upregulated or downregulated in response to *MYC* overexpression or silencing, respectively (Figure S7F, Supporting Information). Moreover, mice bearing KYSE180‐derived xenograft tumors were treated with IGF1R inhibitor Linstinib (25 mg kg^−1^, i.g.), and multiplex IF staining showed lower levels of p‐IGF1R, c‐Myc, and QSOX2 in tumors after treatment with Linstinib than those in control tumors (Figure 6L). In summary, CAFs‐secreted IGF‐1 upregulates QSOX2 expression via the Akt/mTOR/c‐Myc pathway, establishing a positive feedback loop that sustains ESCC cell stemness.

### 2.8 Combining Ebselen, Rapamycin, and Cisplatin Induces Tumor Dormancy

**Figure 7:**
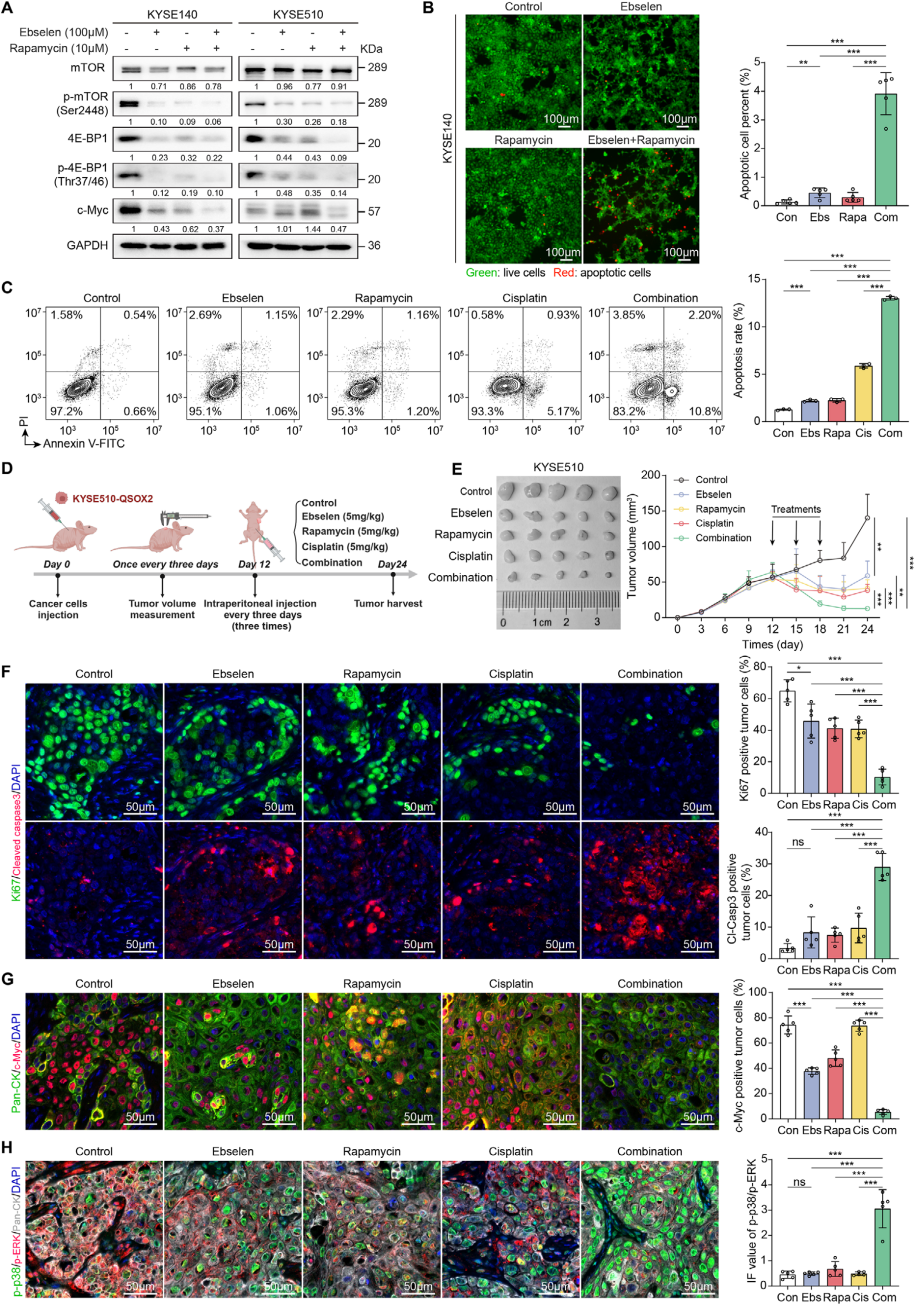
Combining Ebselen, Rapamycin, and Cisplatin inhibits ESCC progression. A) Western blot confirmed that the inhibition of mTOR/4E‐BP1/c‐Myc signaling in KYSE140/KYSE510‐QSOX2 cells by Ebselen (100 µM, 24 h) or/and Rapamycin (10 µM, 24 h) treatments. B) Calcein AM/PI double staining was performed to test the apoptotic cell rate of KYSE140‐QSOX2 cells treated with Ebselen (100 µM, 24 h) or/and Rapamycin (10 µM, 24 h). C) Cell apoptosis assays showed the sensitivity of KYSE510‐QSOX2 cells to Ebselen (100 µM, 24 h), Rapamycin (10 µM, 24 h), Cisplatin (10 µM, 24 h), or their combination. D, E) Tumors were generated by s.c. injection of KYSE510‐QSOX2 cells (3 × 10^6^ cells per mouse). The mice were treated with Ebselen (5 mg kg^−1^, i.p.), Rapamycin (5 mg kg^−1^, i.p.), Cisplatin (5 mg kg^−1^, i.p.) alone or in combination every three days for three times. Concurrently with the treatment, tumor volume was measured. F) Double IF staining showed the percentage of Ki67 or cleaved Caspase‐3 (Cl‐Casp3) positive tumor cells in KYSE510‐QSOX2‐derived xenograft tumors. G) Double IF staining was performed to analyze the percentage of c‐Myc positive tumor cells in KYSE510‐QSOX2‐derived xenograft tumors treated with the indicated treatments. H) Multiplex IF staining showed the mean fluorescence intensity of p‐p38 and p‐ERK in KYSE510‐QSOX2‐derived xenograft tumors after treatments. In B, C, and E panels, data are presented as the mean ± SD; In panels F‐H, data are presented as the mean ± SEM; unpaired two‐tailed Student's t‐test with Welch's correction; *p < 0.05, **p < 0.01, and ***p < 0.001. ns, no significant difference.

Finally, we explored the potential of blocking QSOX2 to suppress ESCC progression in mice. The activation of mTOR/4E‐BP1/c‐Myc signaling in QSOX2‐overexpressed ESCC cells was effectively inhibited by Ebselen treatment in vitro in a time‐ and concentration‐dependent manner (Figure S8A,B, Supporting Information). Although Ebselen can also recognize QSOX1, QSOX1 and QSOX2 are expressed differently in ESCC. IHC staining and gene expression analyses using TCGA and GEO datasets showed higher expression of QSOX2 than QSOX1 in ESCC tissues (Figure S8C–E, Supporting Information). Therefore, Ebselen inhibits ESCC progression mainly by acting on QSOX2. Ebselen combined with Rapamycin markedly downregulated c‐Myc expression in ESCC cells and enhanced cell apoptosis (**Figure** **7**A,B). Moreover, adding Cisplatin to this regimen further increased cell apoptosis (Figure 7C). In vivo xenograft tumor experiments demonstrated that the combined treatment of Ebselen, Rapamycin, and Cisplatin significantly inhibited tumor growth (Figure 7D,E; Figure S8F, Supporting Information). Double IF staining of xenograft tumors showed a decrease in Ki67^+^ tumor cells and an increase in cleaved Caspase 3^+^ tumor cells after combined treatment (Figure 7F). Additionally, combined treatment resulted in a lower percentage of c‐Myc^+^ tumor cells (Figure 7G) and a marked increase in the ratio of phosphorylated p38 (p‐p38) to phosphorylated ERK (p‐ERK) (Figure 7H) than tumors treated alone. A high level of p‐p38/p‐ERK has been demonstrated in dormant tumors.^[^ ^6^, ^28^ ^]^ Therefore, targeting QSOX2‐mTOR feedback loop, in combination with chemotherapy, shows promise in treating ESCC by reducing tumor stemness and promoting tumor dormancy.

## 3 Discussion

The atypical thiol oxidase family includes two QSOX transcript variants, QSOX1 and QSOX2. The human QSOX2 gene, located on chromosome 9q34.3, shares high homology with QSOX1, consists of 12 exons, and encodes a 698‐amino acid protein.^[^ ^29^ ^]^ However, gene expression analyses using TCGA and GEO datasets revealed an inverse expression pattern between QSOX1 and QSOX2 in ESCC. This suggests that QSOX1 and QSOX2 have different expression regulation mechanisms and functions during ESCC progression. QSOX1, a well‐characterized sulfhydryl oxidase, has been shown to protect against oxidative stress‐induced cell death in vitro.^[^ ^30^ ^]^ Our recent study has revealed that high QSOX1 expression in dormant tumors maintains a high oxidation niche, which promotes dormant CSCs immune escape by upregulating the expression of PD‐L1 and itself, and promoting CD8^+^ T‐cell exclusion.^[^ ^5^ ^]^ Therefore, QSOX1 may promote tumor cell survival mainly by regulating the oxidative microenvironment. Overexpression of QSOX2 has been found to be associated with poor survival in patients with non‐small cell lung cancer, colorectal cancer, or prostate cancer.^[^ ^13^, ^14^, ^15^ ^]^ Recent research also revealed that recessive mutations in human QSOX2 can cause growth restriction, gastrointestinal dysmotility, and mild immunodeficiency,^[^ ^31^ ^]^ suggesting that QSOX2 may be related to the development and function of multiple organs in humans. In this study, overexpression of QSOX2 increased the formation of tumor spheres and enhanced the resistance of ESCC cells to Cisplatin and Paclitaxel. In contrast, QSOX2 silence via shRNA significantly suppressed tumor sphere formation in vitro and reduced tumor initiation frequency in vivo. Furthermore, QSOX2 overexpression stimulated the proliferation and metastatic potential of ESCC cells. Overall, these functional analyses demonstrated that QSOX2 enhances tumor stemness and resistance to chemotherapy, contributing to ESCC progression.

Gene co‐expression analysis, overexpression experiments, and IF staining confirmed that c‐Myc is a key mediator of QSOX2‐enhanced ESCC tumor stemness and drug resistance. c‐Myc, a multifunctional transcription factor, significantly influences tumor development and treatment resistance in ESCC by promoting the acquisition of CSC‐like characteristics.^[^ ^32^, ^33^ ^]^ In this study, we demonstrated that high QSOX2 activating mTOR/4E‐BP1/c‐Myc pathway. The mTOR signaling pathway is crucial for maintaining the self‐renewal and differentiation of CSCs, as mTORC1 promotes protein synthesis by phosphorylating substrates such as S6K1 and 4E‐BP1 thereby supporting cell growth and proliferation.^[^ ^34^ ^]^ In CSCs, activation of mTOR signaling preserves the expression of pluripotency‐related genes, including *OCT4*, *NANOG*, and *SOX2*, maintaining stemness.^[^ ^35^ ^]^ Rapamycin, a known mTORC1 inhibitor, reduces CSC self‐renewal and differentiation, thereby suppressing tumor growth and metastasis.^[^ ^34^ ^]^ However, Rapamycin resistance remains a significant challenge.^[^ ^36^ ^]^ This study demonstrated that combining QSOX2 inhibitor Ebselen with Rapamycin further inhibits mTOR activity and c‐Myc expression (Figure 7). This combination represents a promising strategy to suppress tumor stemness in ESCC.

Disulfide bond formation, an important post‐translational modification of proteins, has been recognized as playing a critical role in protein folding and functional maintenance.^[^ ^37^, ^38^ ^]^ Disulfide bond modifications also play a critical role in the initiation and progression of tumors. For example, protein disulfide isomerase (PDI) is highly expressed in various tumors, and inhibition of PDI activity has been shown to suppress tumor cell growth and migration.^[^ ^39^ ^]^ Moreover, PDI enhances DRP1‐S616 phosphorylation by facilitating S‐nitrosylation of DRP1, thereby promoting mitochondrial fission in neurons.^[^ ^40^ ^]^ By regulating the formation and cleavage of disulfide bonds, the activity and stability of proteins can be influenced, thereby affecting tumor cell proliferation and survival.^[^ ^41^ ^]^ A recent study has demonstrated that glucose starvation leads to abnormal accumulation of intracellular disulfide bonds, which induces disulfideptosis in cells with high expression of SLC7A11.^[^ ^42^ ^]^ In this study, we identified that high expression of QSOX2 facilitates disulfide bond formation in TSC2, which subsequently enhances the phosphorylation of TSC2 at Ser939 by Akt in ESCC cells. When TSC2 function is impaired, mTOR/4EBP1/c‐Myc signaling becomes abnormally activated, enhancing tumor stemness.

The regulatory mechanism of QSOX2 expression in cancers remains a mystery. This study demonstrated that the ESCC cells with high expression of QSOX2 were mainly located near the edge of the cancer nest, which was regulated by IGF‐1 secreted by CAFs. IGF‐1 plays a critical role in maintaining CSCs characteristics and facilitating tumor cell proliferation, invasion, and metastasis.^[^ ^43^, ^44^, ^45^ ^]^ Previous studies have shown that IGF‐1 binds to its receptor IGF1R, initiating downstream activation of the Akt/mTOR pathway, thereby promoting tumor cell proliferation and inducing drug resistance.^[^ ^26^, ^46^ ^]^ In this study, we found that IGF‐1 secreted by CAFs upregulated c‐Myc expression to enhance tumor stemness via the IGF1R/Akt/mTOR pathway. Importantly, we also revealed that c‐Myc transcriptionally activated QSOX2 expression, which was upregulated by IGF‐1 stimulation. A recent study has found that administration of recombinant IGF‐1 can complement growth hormone STAT5B dysregulation induced by defective QSOX2 variants and may alleviate multisystem dysfunction.^[^ ^31^ ^]^ These findings suggest that QSOX2 is a key downstream molecule of IGF1R/Akt/mTOR/c‐Myc signaling. Therefore, our study unveils a new feedback loop QSOX2/mTOR that sustains ESCC cell stemness in ESCC.

**Figure 8:**
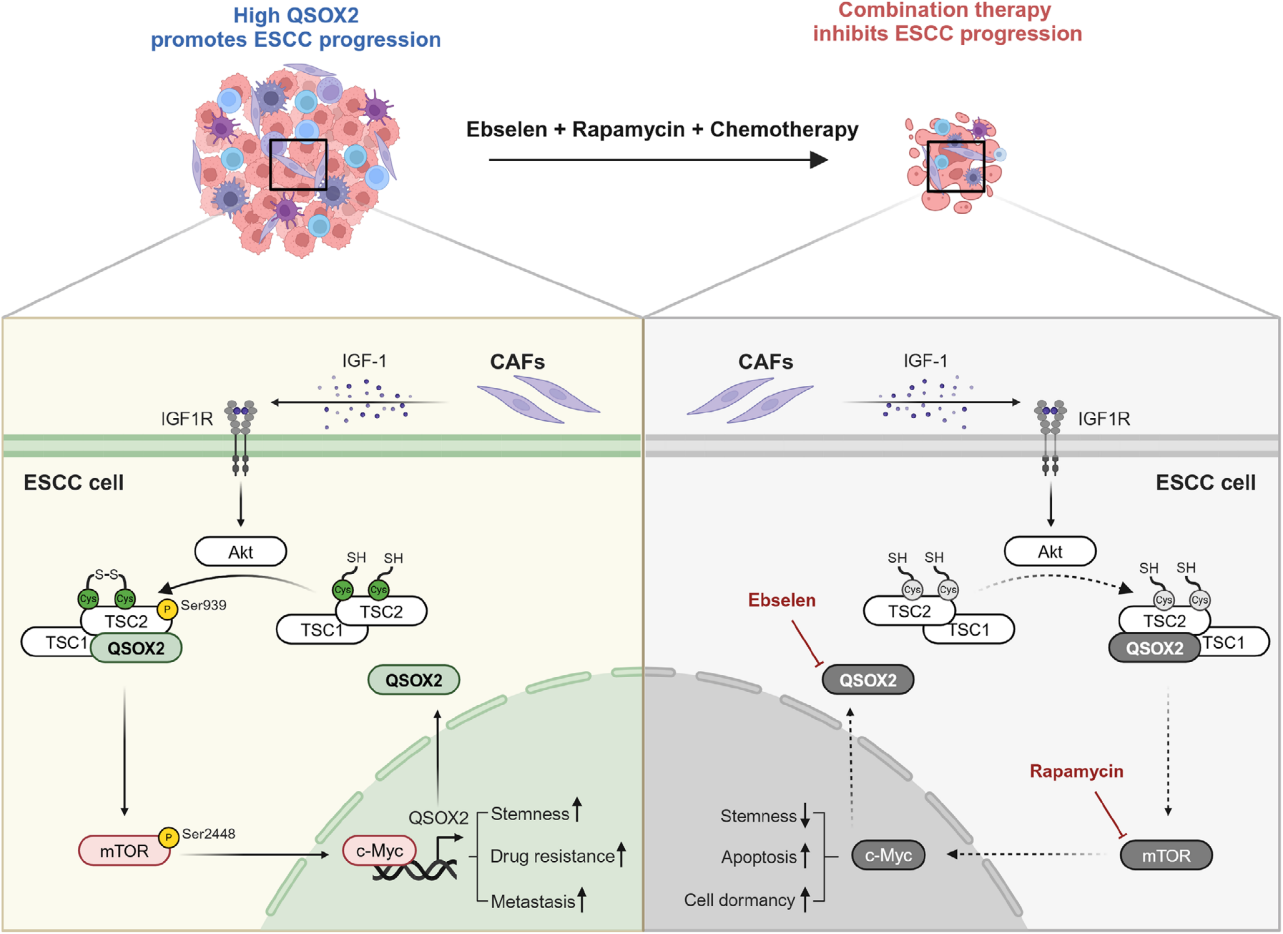
Blocking QSOX2‐mTOR feedback loop, in combination with chemotherapy, reduces tumor stemness and induces tumor dormancy. CAFs secrete IGF‐1 to activate the IGF1R/Akt/mTOR/c‐Myc signaling axis in ESCC cells, leading to an increase in QSOX2 expression. High QSOX2 facilitates the formation of disulfide bonds in TSC2, thereby promoting the binding of TSC2 to Akt and subsequent phosphorylation of TSC2 at the Ser939 site. Phosphorylation of TSC2 at Ser939 relieves its inhibitory effect on mTOR/c‐Myc signaling. Therefore, this mechanism constitutes a positive feedback loop, enhancing tumor stemness, chemotherapy drug resistance, and metastasis of ESCC cells. Inhibiting QSOX2 with Ebselen, in combination with Rapamycin and chemotherapy, inhibits ESCC progression by blocking QSOX2‐mTOR feedback loop, suppressing tumor stemness, enhancing chemotherapy sensitivity, and promoting tumor dormancy.

In summary, we demonstrated that CAFs‐secreted IGF‐1 upregulates QSOX2 expression by activating IGF1R/Akt/mTOR/c‐Myc signaling axis. High QSOX2 further promotes disulfide bond formation in TSC2, which facilitates the phosphorylation of TSC2 at Ser939 by Akt, leading to activation of mTOR pathway in ESCC cells. This positive feedback loop enhances ESCC stemness, drug resistance, and metastasis. Notably, the combination of QSOX2 inhibitor Ebselen, mTOR inhibitor Rapamycin, and chemotherapy effectively inhibits tumor growth, reduces tumor stemness, and induces tumor dormancy in mouse (**Figure** **8**). These findings suggest a potential therapeutic strategy for ESCC patients with high QSOX2 expression.

## 4 Experimental Section

### Clinical Samples and Cell Lines

Primary ESCC tissues and their adjacent normal esophageal tissues used for western blot and immunostaining were obtained from Linzhou Cancer Hospital (Linzhou, China). All clinical samples used in this study were approved by the Committees for Ethical Review at the Sun Yat‐sen Memorial Hospital (Guangzhou, China) (#SYSKY‐2023‐670‐01). Written informed consent was obtained from all patients or their legal representatives for the use of their tissue samples in this study. The human immortalized esophageal epithelial cell line (HET‐1A) and six ESCC cell lines (KYSE30, KYSE140, KYSE150, KYSE180, KYSE410, and KYSE510) were purchased from the DSMZ (Braunschweig, Germany). The human embryonic kidney 293FT cell line was purchased from the ATCC (Manassas, VA). The mouse esophageal cancer cell line mEC2 used in this study was donated by Prof. Li Fu at Shenzhen University (Shenzhen, China).^[^ ^47^ ^]^ All cells were cultured in DMEM (GIBCO, #C11995500BT) supplemented with 10% FBS (ExCell Bio, #FSP500). All cells were maintained at 37 °C with 5% CO_2_.

### Plasmids and Transfection

Overexpression or shRNA plasmids of *QSOX2* were ordered from Genecopoeia. The sequences of the two shRNAs targeting *QSOX2* are as follows: 5′‐GGCTATTGTCTTTGAAAGCAA‐3′ and 5′‐GCGGATTTCTGGAATATTCCT‐3′. The lentiviral plasmids were transfected into 293FT cells for virus production using PEI MAX‐Transfection Grade Linear Polyethylenimine (Polysciences, #24765‐100). After 48–72 h, the virus‐containing supernatant was collected and filtered through a 0.45 µm filter (Pall, #PN4614‐1EA). ESCC cells were infected with the collected virus‐containing supernatants.

### Animal Experiments

BALB/c‐Nude mice (Strain NO. D000521) were purchased from GemPharmatech (Nanjing, China) and were housed in a barrier facility at the Experimental Animal Center, Sun Yat‐sen Memorial Hospital. All animal research was conducted in accordance with protocols approved by the Institutional Animal Care and Use Committee of Sun Yat‐sen Memorial Hospital (#AP20240062). To establish the subcutaneous xenograft tumor model in nude mice, KYSE180 (1 × 10^6^) or KYSE510 (3 × 10^6^) cells from different treated groups, resuspended in DMEM, were subcutaneously injected into the flanks of each mouse. Tumor volume (mm^3^) was calculated using the formula: Volume = 0.5 × Length × Width^2^. To block IGF1R, mice bearing KYSE180 tumors were gavaged daily with 100 µL of Linsitinib (25 mg kg^−1^, Selleck, #S1091) starting on day 8 after tumorigenesis. Control mice were gavaged with 100 µL of a solvent containing 5% DMSO, 40% PEG300, 5% Tween‐80, and 50% saline. In the drug treatment experiments, when the tumors reached 50 mm^3^, the mice were randomly divided into treatment and control groups. The treatment groups were injected with Cisplatin (5 mg kg^−1^), Rapamycin (5 mg kg^−1^), Ebselen (5 mg kg^−1^) alone or in combination every three days, while the control group received vehicle injections. At the end of the experiment, the tumors were collected for histological and immunofluorescence staining. To establish an experimental lung metastasis model, 1.5 × 10^6^ ESCC cells were injected through the tail vein. Three months after injection, the mice were sacrificed, and the whole lung from each mouse was removed, fixed with 4% paraformaldehyde, embedded in paraffin, and serially sectioned. Paraffin sections were stained with hematoxylin‐eosin, and the number of metastatic lesions in the lungs was counted under a microscope.

### Western Blot

Total proteins were extracted from ESCC cells and tumor tissues using RIPA lysis buffer, separated by 6% or 10% sodium dodecyl sulfate polyacrylamide gel electrophoresis (SDS‐PAGE), and electro‐transferred onto a polyvinylidene fluoride (PVDF) membrane. After being blocked with 5% BSA for 1 h at room temperature, the membrane was incubated with the primary antibody overnight at 4 °C. Subsequently, the membrane was washed three times with TBST and incubated with the secondary antibody at room temperature for 1 h. Finally, the membrane was washed three additional times with TBST. Primary antibodies and their dilutions are listed in Table S1 (Supporting Information). The immunoreactive bands were developed using an ultrasensitive ECL detection reagent (Meilunbio, #MA0186‐2).

### Flow Cytometry

ESCC cells with QSOX2 overexpression or knockdown were seeded into 6‐well plates. The cells were cultured for 24 h, harvested, and washed with cold PBS before being stained with APC‐conjugated anti‐CD271 (Biolegend, #345108) for 30 min. Subsequently, the cells were washed again with cold PBS and analyzed by flow cytometry (Beckman Cytoflex LX).

### Immunofluorescence (IF) Staining

IF staining of tumor cells was performed for three purposes: to investigate the localization of QSOX2, TSC1, and TSC2; to analyze Akt level in Vector‐ or QSOX2‐labeled tumor cells; and to evaluate changes in signaling pathways after treatment with IGF‐1 (10 ng mL^−1^, PeproTech, #100‐11‐100) or/and Linsitinib (5 µM, Selleck, #S1091) for 24 h. Tumor cells were pre‐seeded on glass slides in a 24‐well plate and treated as required based on the experimental aim. The cells were then washed twice with PBS and fixed with 2% paraformaldehyde (Biosharp, #BL539A) at room temperature for 15 min. Next, the cells were permeabilized with 0.1% Triton X‐100 (Beyotime, #P0096) at room temperature for 10 min. After being blocked with 5% BSA solution at room temperature for 30 min, the cells were incubated with primary antibodies and subsequently with secondary antibodies. The primary antibodies and their dilutions are listed in Table S2 (Supporting Information).

### Immunohistochemistry (IHC) Staining

After 24 h of fixation with 4% paraformaldehyde solution (Biosharp, #BL539A), the tissues were dehydrated, paraffin‐embedded, and sectioned into 5 µm slices. The paraffin‐embedded tissues were dewaxed with xylene, hydrated using a gradient of alcohol concentrations (100%, 95%, 85%, 75%, and 50%), and subjected to microwave treatment for antigen retrieval. Once the slices cooled to room temperature, they were soaked in 3% H_2_O_2_ for 10 min to block endogenous peroxidase activity.

Subsequently, the primary antibodies (Table S2, Supporting Information) were incubated overnight at 4 °C. Following three washes with PBS, the secondary antibody (ZS, #PV‐6000‐55) was incubated at 37 °C for 30 min. The DAB Substrate Kit (ZS, #ZLI‐9017) was used to visualize the target proteins, which were observed under a light microscope. Multiple IF staining was performed using the Multiplex IHC Kit (Panovue, #10001100020) according to the manufacturer's instructions. Elution between different primary antibodies was performed using an antibody eluate (Absin, #abs994). After the final marker was labeled, the tissue sections were mounted with an antifade mounting solution containing DAPI (Beyotime, #P0131). Images were acquired using a laser confocal microscope (Olympus).

### Sphere Formation Assay

The culture medium for sphere formation comprised 1× DMEM/F12 medium (Gibco, #10565018), 0.5% methylcellulose (Sigma, #M0512), 1× B27 supplement (Gibco, #17504‐044), 20 ng mL^−1^ EGF (PeproTech, #AF‐100‐15), 10 ng mL^−1^ FGF‐basic (PeproTech, #100‐18B), 4 µg mL^−1^ Insulin (Selleck, #S6955), and 100 U mL^−1^ Penicillin‐Streptomycin solution (NCM, #C100C5). Following cell counting, 1×10^4^ tumor cells in 1 mL^−1^ of the prepared culture medium were gently seeded into each well of an ultra‐low attachment 24‐well plate (LV‐Biotech, #LV‐ULA002‐12 W). The culture plate was placed in a 37 °C cell incubator with 5% CO_2_. After three days of incubation, images were captured using a light microscope, and the number of spheres was counted to assess the stemness level of the tumor cells.

### Calcein AM/PI Double Staining

The Calcein AM/PI double staining kit (Elabscience, #E‐CK‐A354) enabled simultaneous double fluorescence staining of live and dead cells. Adherent cells and spheres were treated with the drug as required for 24 h. The medium was then removed, and the cells were washed once with PBS. The Calcein AM/PI mixture was added to stain the cells for 15 min, followed by examination and imaging using a fluorescence microscope. Five visual fields were selected per group for statistical analysis.

### Cell Apoptosis Assays

Tumor cells were pre‐implanted in 6‐well plates. After the cells were attached, 20 µM Paclitaxel (YuanYe, #B20341), 10 µM Cisplatin (Selleck, #S1166), 10 µM Rapamycin (Selleck, #S1039), or 100 µM Ebselen (Selleck, #S6676) were added to the cells for 24 h as required. The cells were then digested with 0.25% trypsin‐EDTA and collected by centrifugation. Subsequently, the cells were stained with an Annexin V‐FITC/PI apoptosis detection kit (Elabscience, #E‐CK‐A211) for 15 min, and immediately analyzed using flow cytometry (Beckman Cytoflex LX).

### IC50 Assay

ESCC cells with QSOX2 overexpression or knockdown were seeded into 96‐well plates at a density of 2000 cells per well. After 12 h of seeding, the cells were treated with a gradient of Cisplatin concentrations for 48 h. The medium was then removed, and 10 µL of CCK‐8 solution (APExBIO, #K1018) and 90 µL of complete medium were added to each well. After incubating the cells for 2 h, absorbance was measured at 450 nm using an absorbance meter (BioTek Synergy H1). IC50 values were calculated using GraphPad Prism 8.0 software.

### EdU Staining

EdU staining was performed using the Cell‐Light EdU Apollo643 In Vitro Kit (RIBOBIO, #C10310‐2). Glass slides were pre‐placed in 24‐well plates, and 2 × 10^4^ tumor cells were seeded into each well. When the cells reached the logarithmic growth phase, EdU solution was added to each well at a ratio of 1:1000, and the cells were incubated for 2 h. The cells were then washed twice with PBS for 5 min each and fixed with 2% paraformaldehyde for 15 min. After removing the liquid, 2 mg mL^−1^ glycine was added, and the cells were incubated for 5 min to neutralize excess aldehyde groups. After washing with PBS, the cells were permeabilized with 0.5% Triton X‐100 in PBS for 10 min. Following another PBS wash, Apollo staining solution was applied, and the cells were incubated at room temperature for 30 min. After discarding the staining solution, the cells were washed three times with 0.5% Triton X‐100 in PBS, each wash lasting 10 min. Finally, the slides were mounted with DAPI‐containing medium and covered with a coverslip. The slides were then observed under a confocal microscope (Olympus).

### Cell Migration and Invasion Assays

Cell motility was assessed using cell migration and invasion assays with transwell chambers (8 µm pore size) with or without a Matrigel membrane (Corning, USA). The Matrigel membrane was pre‐coated with 50 µL of BD Matrigel mixture (diluted 1:8 in DMEM) at 37 °C for 1 h prior to the assay. Briefly, after serum starvation for 24 h, cells (5 × 10^4^ for KYSE30; 1 × 10^5^ for KYSE140, KYSE180, and KYSE510) suspended in 200 µL of serum‐free DMEM were seeded into the upper chamber, while 500 µL of medium containing 10% FBS was added to the lower chamber. The abilities of cell migration or invasion were assessed after 48 h of culture for KYSE30 cells and 72 h for KYSE140, KYSE180, and KYSE510 cells. After removing cells from the upper surface of the filter using a cotton swab, the migrative or invasive cells attached to the lower surface of the membrane were fixed with 4% paraformaldehyde for 15 min, stained with 0.1% crystal violet for 15 min, and quantified by counting the cells in five random fields under a microscope.

### Immunoprecipitation (IP) Assay and Mass Spectrometry Analysis

The proteins were processed and obtained using a Pierce Classic Magnetic IP/Co‐IP Kit (Thermo Scientific, #88 804) prior to mass spectrometry. Briefly, cell lysates from KYSE30 and KYSE180 cells were subjected to IP assay using a QSOX2 antibody. Proteins interacting with QSOX2 were enriched with magnetic beads and subsequently underwent washing, reduction, alkylation, enzymatic digestion, peptide extraction, and desalting to prepare samples for mass spectrometry analysis. The samples were then analyzed for proteomics using the Nalc 1200‐FAIMS Fusion Orbitrap mass spectrometer. Proteins identified in both cell lines were considered high‐potential QSOX2 binding partners. Additionally, whole‐cell lysates from cell lines overexpressing QSOX2 were subjected to IP assay with a TSC2 antibody. The resulting protein complexes were boiled in loading buffer, and the binding of TSC2 to QSOX2 and Akt was assessed by western blot.

### 3D Structural Analysis

Structural data of QSOX2, Akt, TSC1, and TSC2 proteins used for protein‐protein docking were obtained from the AlphaFold Protein Structure Database. Molecular docking was performed using HDOCK server with a hybrid algorithm that integrates template‐based modeling with ab initio free docking. The 3D structures of protein interaction were visualized using PyMOL molecular visualization software.

### Chromatin Immunoprecipitation Coupled with Quantitative Polymerase Chain Reaction (ChIP‐qPCR)

ChIP was performed using the SimpleChIP Enzymatic Chromatin IP Kit (Cell Signaling Technology, #9003). Briefly, the cells were cross‐linked with 1% formaldehyde. The DNA was then digested using micrococcal nuclease included in the kit, followed by IP assay with anti‐c‐Myc (Cell Signaling Technology, #18583) or rabbit IgG (Cell Signaling Technology, #2729) as a control. Immunoprecipitated and eluted DNA was purified using columns and amplified by qPCR with the primers (forward: 5′‐CGCACGTGACGGTGG‐3′, reverse: 5′‐CGGCGCGCTGAACTT‐3′).

### Statistical Analyses

GraphPad Prism (version 8.0) was used for statistical analysis and graph preparation. An unpaired *t*‐test was used to compare the differences between the two groups. Pearson correlation analysis was used to calculate the correlation between the expression levels of the two genes. Kaplan‐Meier analysis and log‐rank test were used for calculation. The experimental results were considered statistically significant when *p* < 0.05.

## Conflict of Interest

The authors declare no competing interests.

## Author Contributions

W.‐M. C., X.‐P. Z., X. S., and H.‐C. L. contributed equally to this article. L. L. designed research; W.‐M. C., X.‐P. Z., X. S., H.‐C. L., and Y.‐Y. Y. performed research; X. W., Y. L., Y. F., and Z. C. analyzed data; Q. Y., C. J., and Y. J. provided technical support; and W.‐M. C. and X.‐P. Z. wrote the paper. All authors reviewed and approved the manuscript.

## Supporting information



Supporting Information

## Data Availability

The data that support the findings of this study are available in the supplementary material of this article.
