# Supplementary material for: QSOX2‐Mediated Disulfide Bond Modification Enhances Tumor Stemness and Chemoresistance by Activating TSC2/mTOR/c‐Myc Feedback Loop in Esophageal Squamous Cell Carcinoma
Source: Adv Sci (Weinh). 2025 May 28;12(31):e00597. doi: 10.1002/advs.202500597 (PMC12376698; doi:10.1002/advs.202500597)
Supplement: Supplementary file 1 — Supporting Information [file ADVS-12-e00597-s001.docx]

**Supporting Information**

**QSOX2-mediated disulfide bond modification enhances tumor stemness and chemoresistance by activating TSC2/mTOR/c-Myc feedback loop in esophageal squamous cell carcinoma**

**Including:**

- **Supporting Figures and Figure Legends**

Figure S1-S8.

- **Supporting Tables**

Table S1-S4.


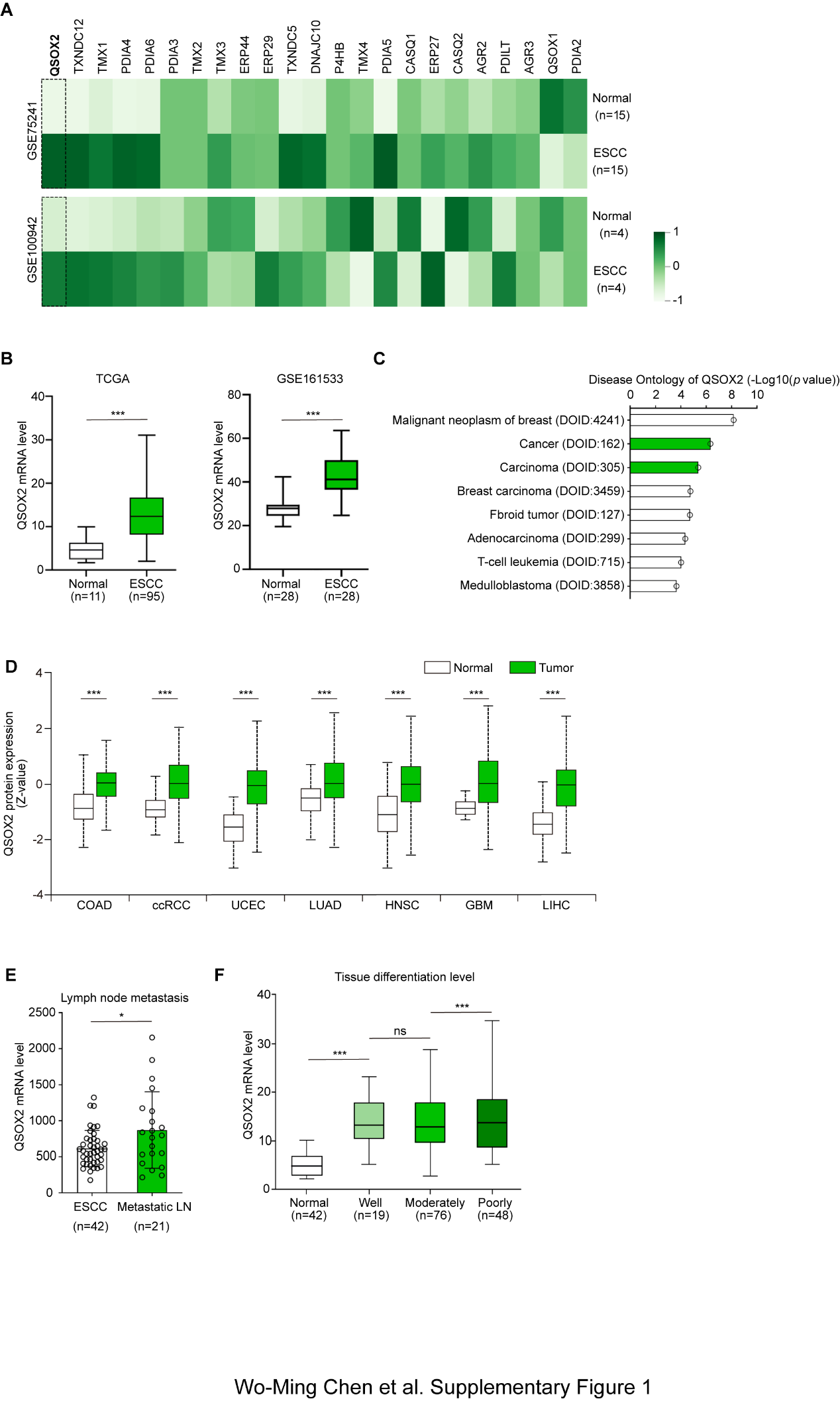


**Fig. S1 QSOX2 is overexpressed in ESCC and correlates with poor differentiation.**

**A.** Heatmap analysis revealed a significant upregulation of *QSOX2* expression in ESCC tumor tissues compared with adjacent normal esophageal epithelium tissues.

**B.** *QSOX2* mRNA levels in ESCC and normal tissues were analyzed based on TCGA database and GEO dataset (GSE36376).

**C.** Disease ontology of *QSOX2*.

**D.** QSOX2 protein levels (UALCAN) in primary tumors and normal tissues. COAD, Colon Cancer; ccRCC, Clear cell renal cell carcinoma; UCEC, Uterine Corpus Endometrial Carcinoma; LUAD, Lung adenocarcinoma; HNSC, Head and Neck squamous cell carcinoma; GBM, Glioblastoma multiforme; LIHC, Liver hepatocellular carcinoma.

**E.** Expression levels of QSOX2 in ESCC and metastatic lymph node were analyzed based on TCGA database. LN, Lymph node.

**F.** *QSOX2* mRNA expression in ESCC tissues at different differentiation levels.

In all panels, data are presented as the mean ± SD; unpaired two-tailed Student’s *t*-test with Welch’s correction; **P* < 0.05, ****P* < 0.001. ns, no signiﬁcant difference.


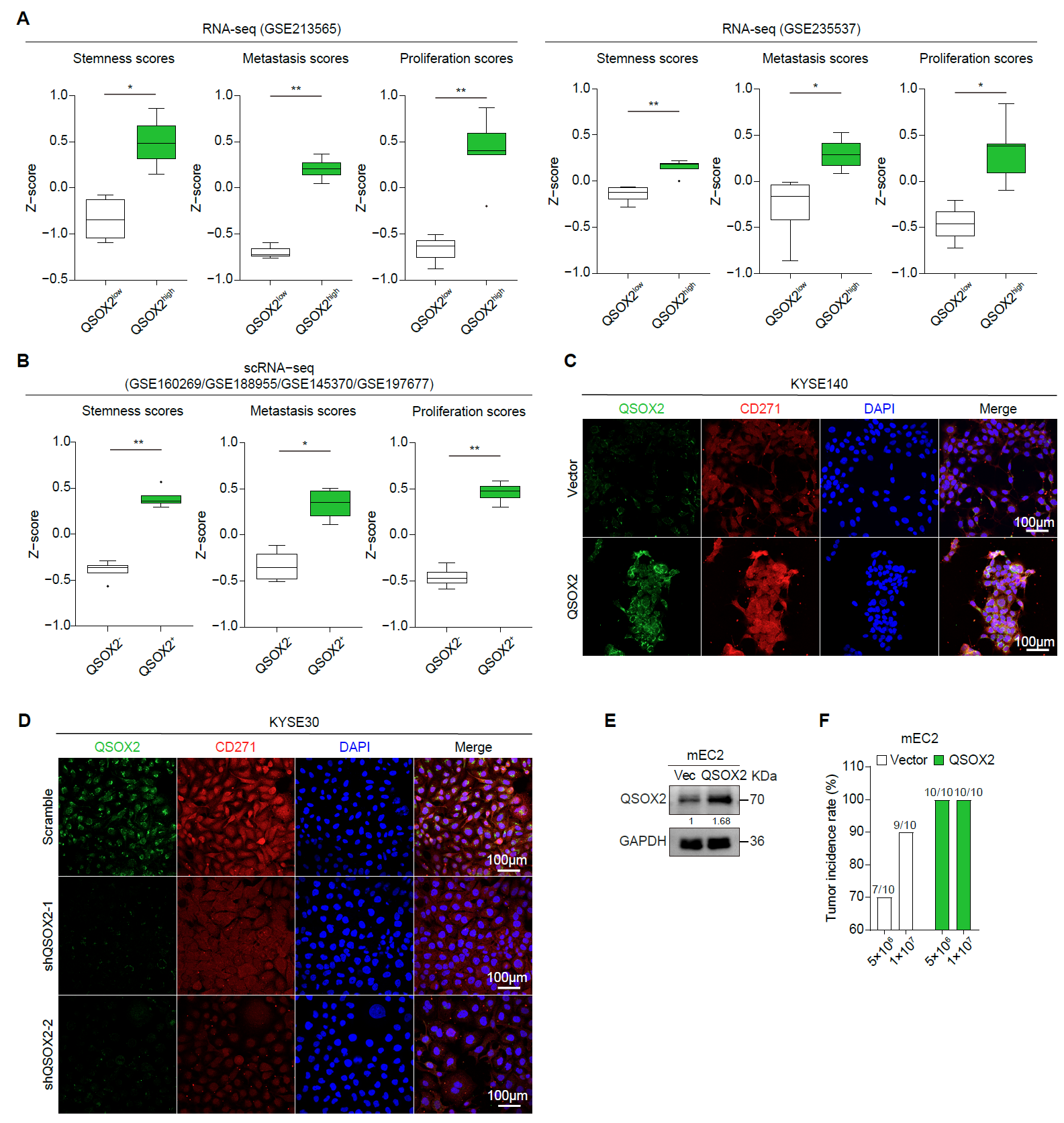


**Fig. S2 QSOX2 enhances tumor stemness of ESCC cells.**

**A.** RNA-Seq data (GSE213565 and GSE235537) analysis revealed differential scores for stemness, proliferation, and metastasis markers between QSOX2-high and QSOX2-low expressing samples.

**B.** scRNA-Seq data (GSE160269, GSE188955, GSE145370 and GSE197677) analysis revealed differential scores for stemness, proliferation, and metastasis markers between QSOX2^+^ and QSOX2^-^ ESCC cells.

**C.** Double IF staining confirmed the upregulation of CD271 after QSOX2 overexpression in KYSE140 cells.

**D.** Double IF staining showed the downregulation of CD271 after QSOX2 silence in KYSE30 cells.

**E.** Western blot was performed to conﬁrm the overexpression of QSOX2 in ESCC cells mEC2.

**F.** Tumor incidence was evaluated in C57BL/6 mice one month after injection of mEC2 cells with a gradient cell count.

In panels **A** and **B**, data are presented as the mean ± SD; unpaired two-tailed Student’s *t*-test with Welch’s correction; **P* < 0.05, ***P* < 0.01.


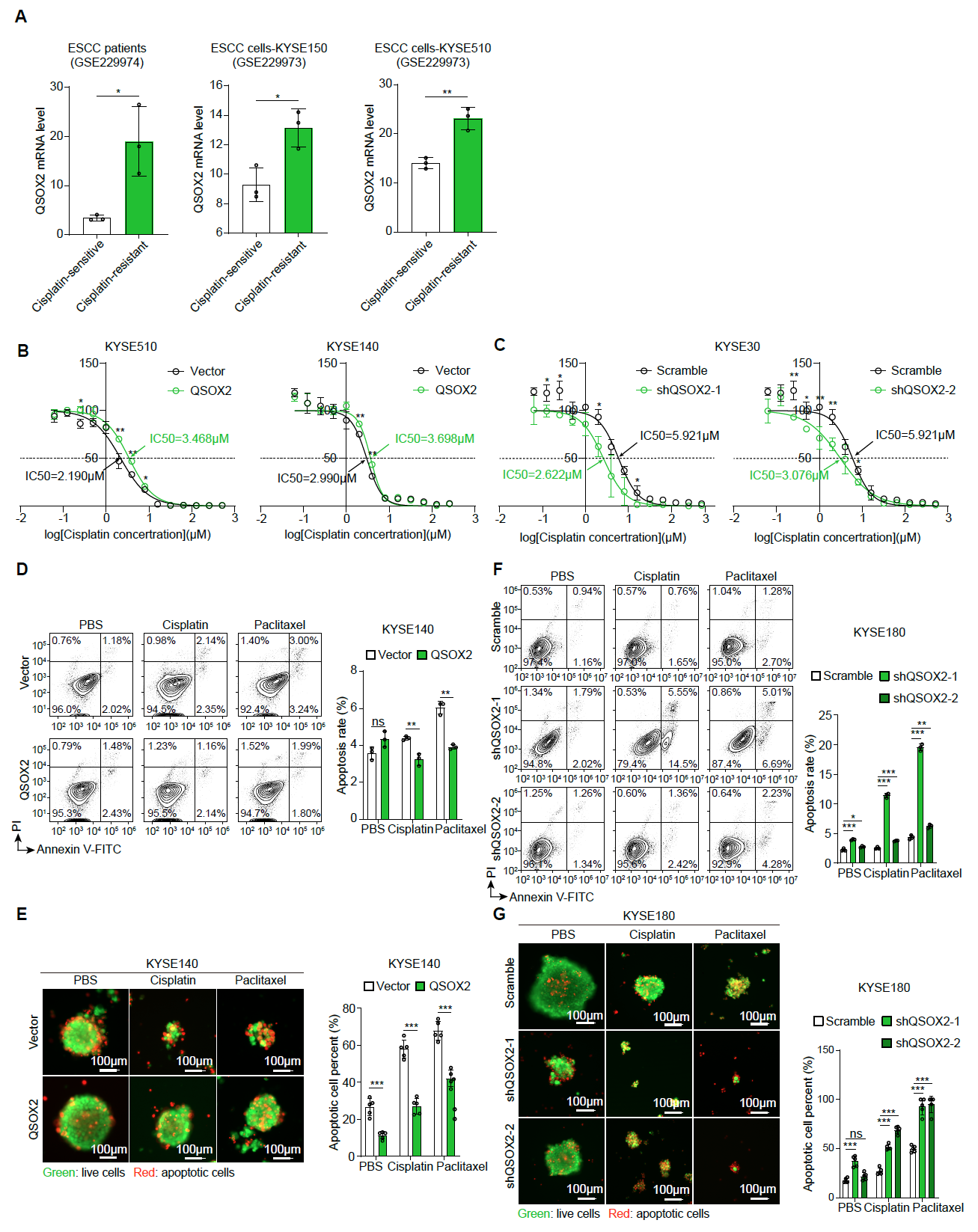


**Fig. S3 QSOX2 promotes chemotherapy drug resistance of ESCC cells.**

**A.** QSOX2 mRNA expression in cisplatin-sensitive and -resistant ESCC patient tissues or cell lines were analyzed based on GEO datasets.

**B.** IC50 values of KYSE140/KYSE510-Vector and KYSE140/KYSE510-QSOX2 cells under treatment of Cisplatin (48 h).

**C.** IC50 values of KYSE30-scramble and KYSE30-shQSOX2-1/2 cells after treatment with Cisplatin (48 h).

**D.** Cell apoptosis assays assessing the sensitivity of ESCC cells with QSOX2 overexpression to Cisplatin or Paclitaxel.

**E.** Calcein AM/PI double staining was performed to test the apoptotic cell rate of ESCC cells with QSOX2 overexpression after treatment with Cisplatin or Paclitaxel.

**F.** Cell apoptosis assays assessing the sensitivity of ESCC cells with QSOX2 silence to Cisplatin or Paclitaxel.

**G.** Calcein AM/PI double staining was performed to test the apoptotic cell rate of ESCC cells with QSOX2 silence after treatment with Cisplatin or Paclitaxel.

In all panels, data are presented as the mean ± SD; unpaired two-tailed Student’s *t*-test with Welch’s correction; **P* < 0.05, ***P* < 0.01, and ****P* < 0.001. ns, no signiﬁcant difference.


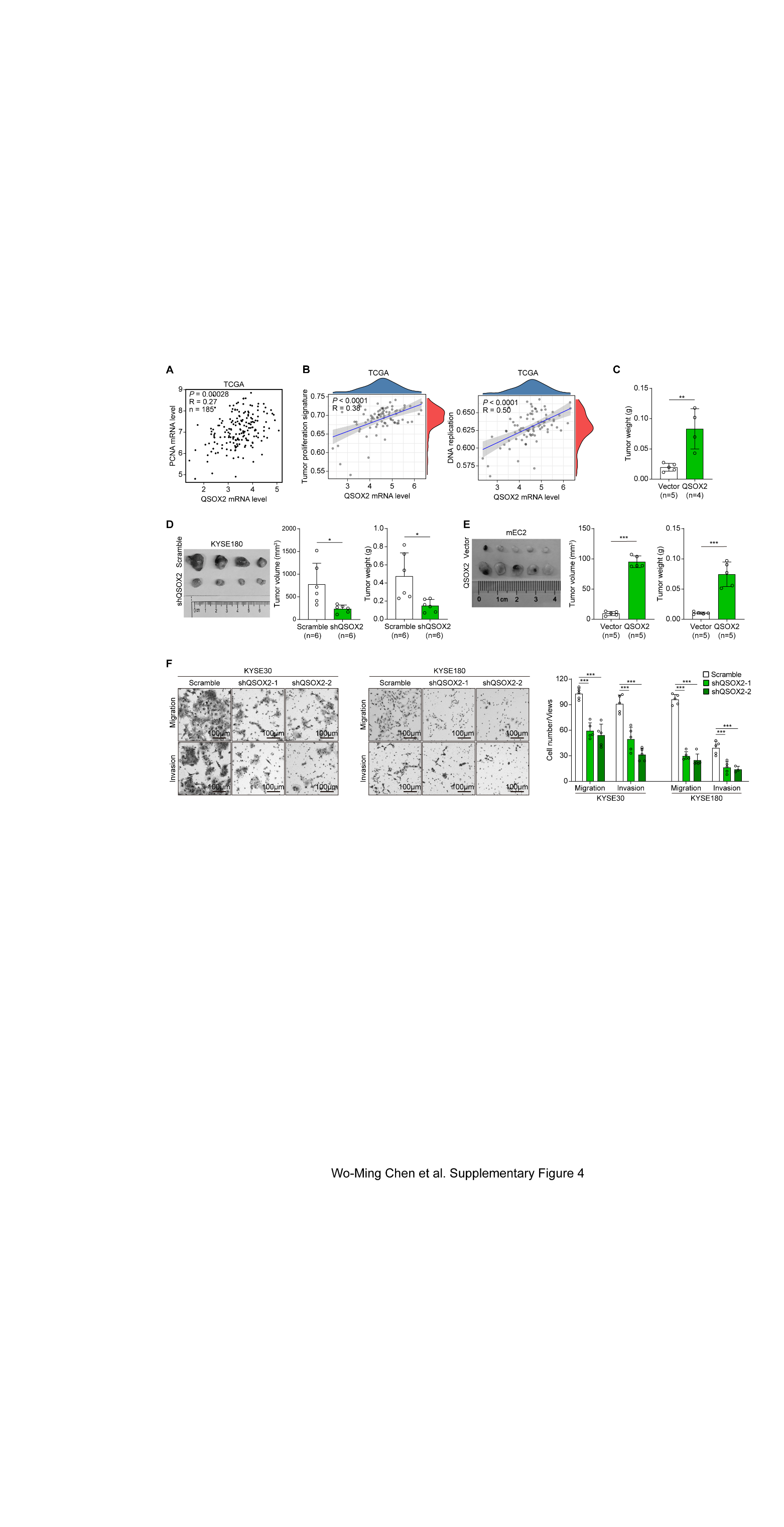


**Fig. S4 QSOX2 promotes proliferation and metastasis of ESCC cells.**

**A.** Co-expression analysis between QSOX2 and PCNA in ESCA using TCGA cohort.

**B.** Spearman correlation analysis showing the positive correlation between tumor proliferation or DNA replication pathway score and QSOX2 expression using TCGA cohort.

**C.** Xenograft tumor weight from KYSE510-Vector and KYSE150-QSOX2 were calculated.

**D.** Xenograft tumor experiment was performed using KYSE180-Scramble and KYSE180-shQSOX2 cells. Tumor volume and weight were calculated.

**E.** Xenograft tumor experiment was performed using mEC2-Vector and mEC2-QSOX2 cells. Tumor volume and weight were calculated.

**F.** Transwell assay assessing the cell migration and invasion abilities of KYSE30/KYSE180-Scramble and KYSE30/KYSE180-shQSOX2 cells.

In all panels, data are presented as the mean ± SD; unpaired two-tailed Student’s *t*-test with Welch’s correction; **P* < 0.05, ***P* < 0.01, and ****P* < 0.001.


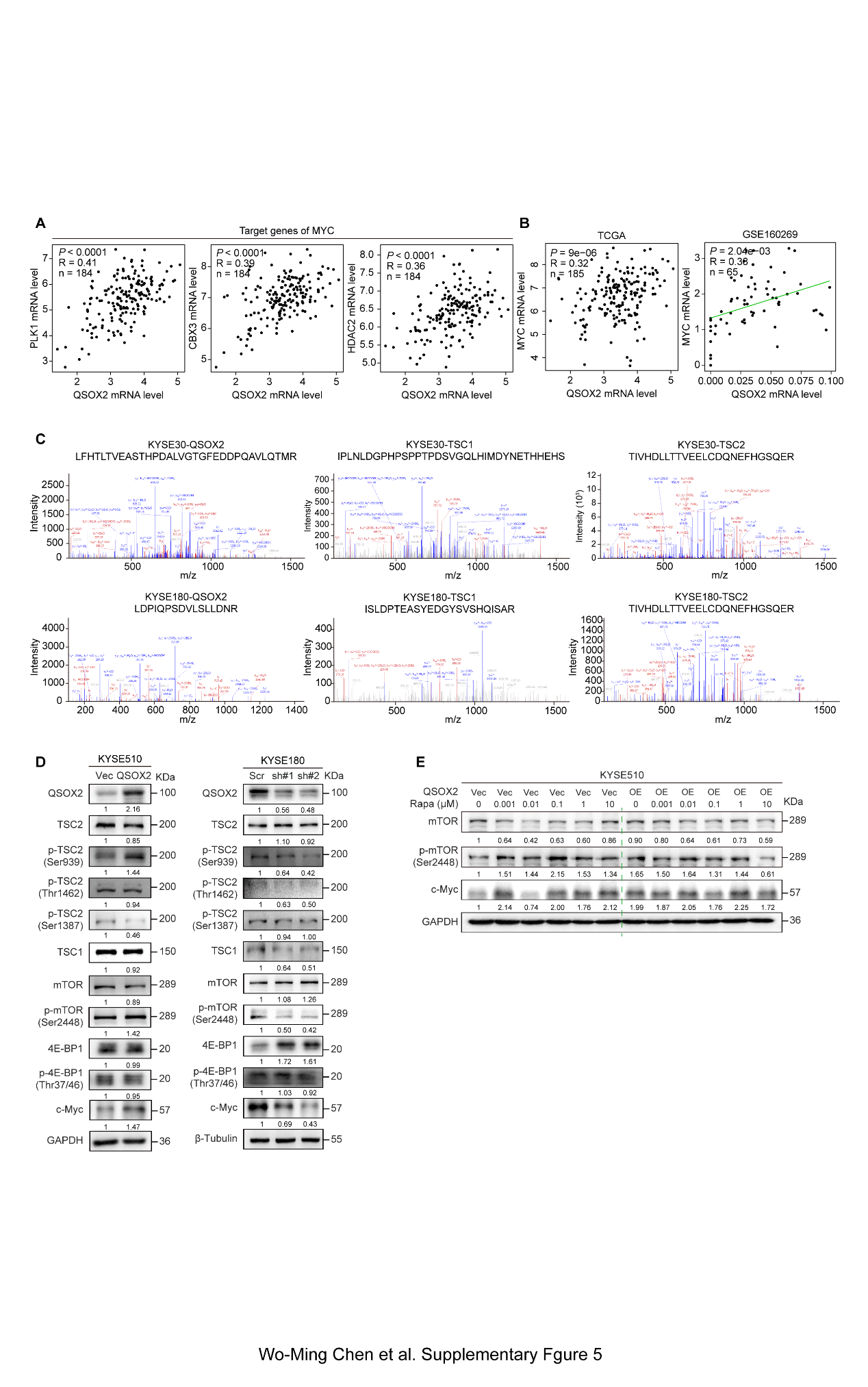


**Fig. S5 QSOX2 activates the mTOR/c-Myc signaling by enhancing p-TSC2^Ser939^.**

**A.** *QSOX2* and target genes of *MYC* (*PLK1*, *CBX3* and *HDAC2*) co-expressed in ESCA in the TCGA cohort.

**B.** Co-expression analysis between *QSOX2* and *MYC* in ESCA using TCGA cohort and GEO dataset (GSE160269).

**C.** LC-MS/MS analysis of QSOX2 binding proteins in KYSE30 and KYSE180 cells.

**D.** Western blot showing the activation or inactivation of TSC2/mTOR/4E-BP1/c-Myc signaling after QSOX2 overexpression or silence in ESCC cells.

**E.** Western blot was performed to test the levels of p-mTOR^Ser2448^ and c-Myc in KYSE510-Vector and KYSE510-QSOX2 cells treated with different concentrations of Rapamycin (24 h).


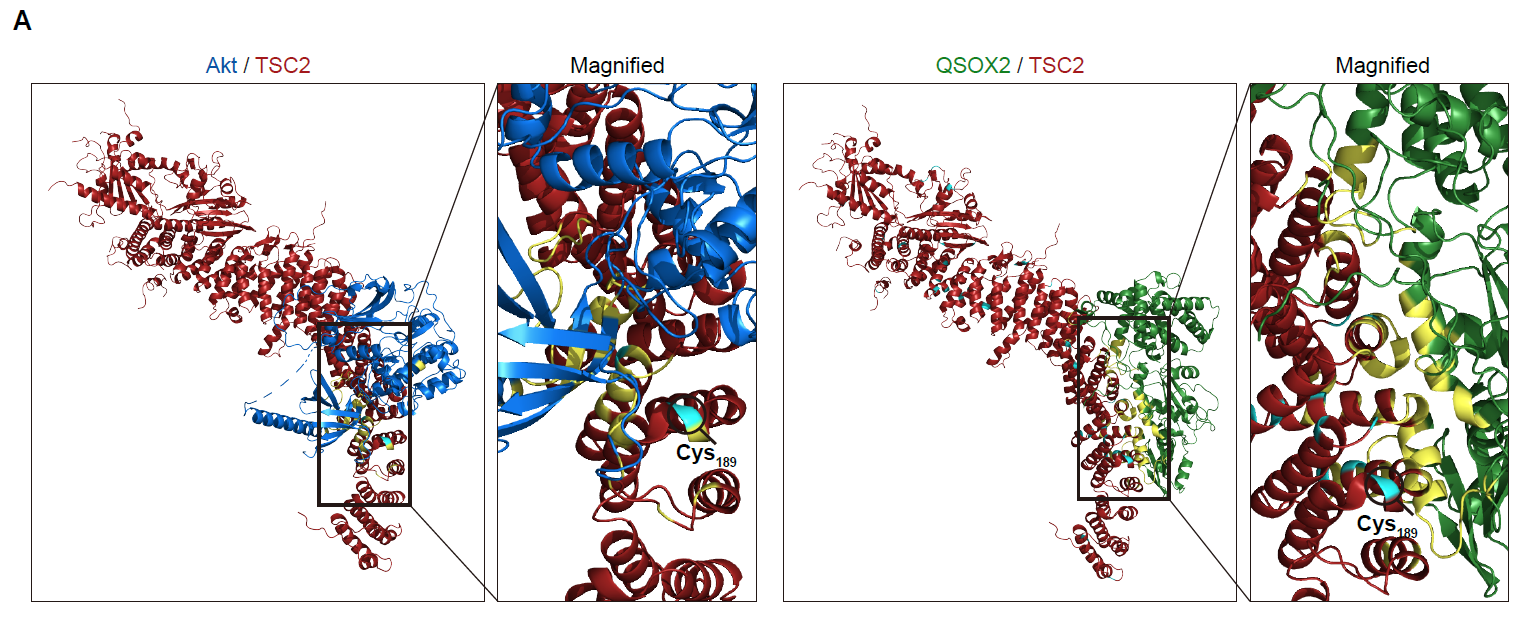


**Fig. S6 QSOX2 promotes disulfide bonds formation of TSC2 by binding Akt.**

A. Molecular docking experiments revealed the QSOX2/TSC2 and Akt/TSC2 complexes. QSOX2, green; Akt, blue; TSC2, red; Protein binding site, yellow; Cysteine, cyan.


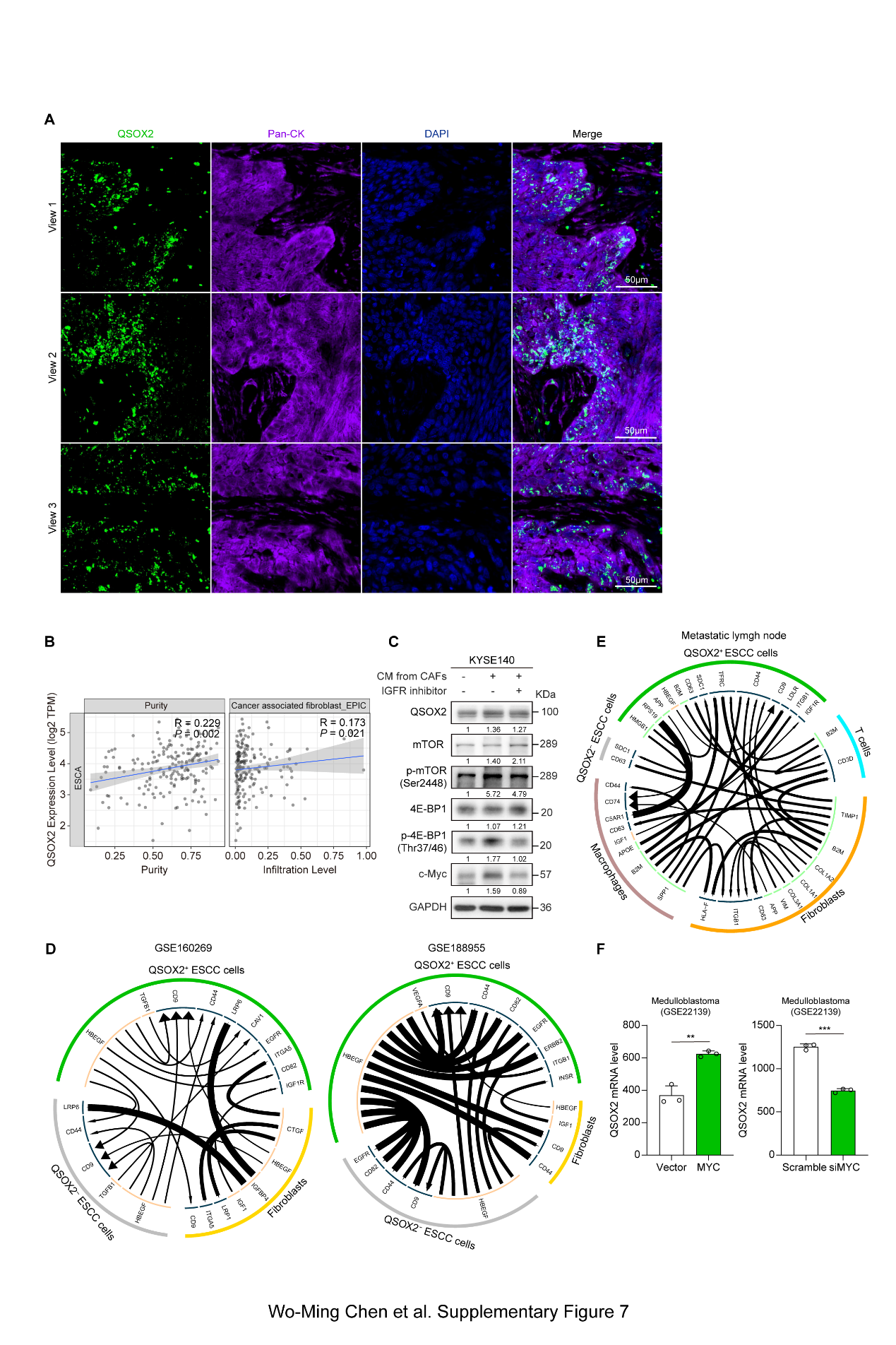


**Fig. S7 IGF-1 upregulates QSOX2 expression via c-Myc.**

**A.** Double IF staining showed that QSOX2 was highly expressed at the edge of tumor nests.

**B.** Correlation between QSOX2 and Cancer associated fibroblast EPIC in ESCA

**C.** Western blot confirmed the activation of IGF1R/Akt/mTOR/c-Myc/QSOX2 signaling in KYSE140 cells by CAFs-conditioned media and the stimulation was inhibited by Linsitinib treatment (5 μM, 24 h).

**D.** CellChat analysis in ESCC identified significant interactions between QSOX2-negative/-positive tumor cells and CAFs.

**E.** CellChat analysis in metastatic lymph nodes of ESCC identified significant interactions between QSOX2-positive tumor cells and various microenvironmental cell populations.

**F.** *QSOX2* mRNA expression in medulloblastoma cells after MYC overexpression or silence was analyzed using GEO dataset (GSE22139).

In panel **F**, data are presented as the mean ± SD; unpaired two-tailed Student’s *t*-test with Welch’s correction; ***P* < 0.01, and ****P* < 0.001.


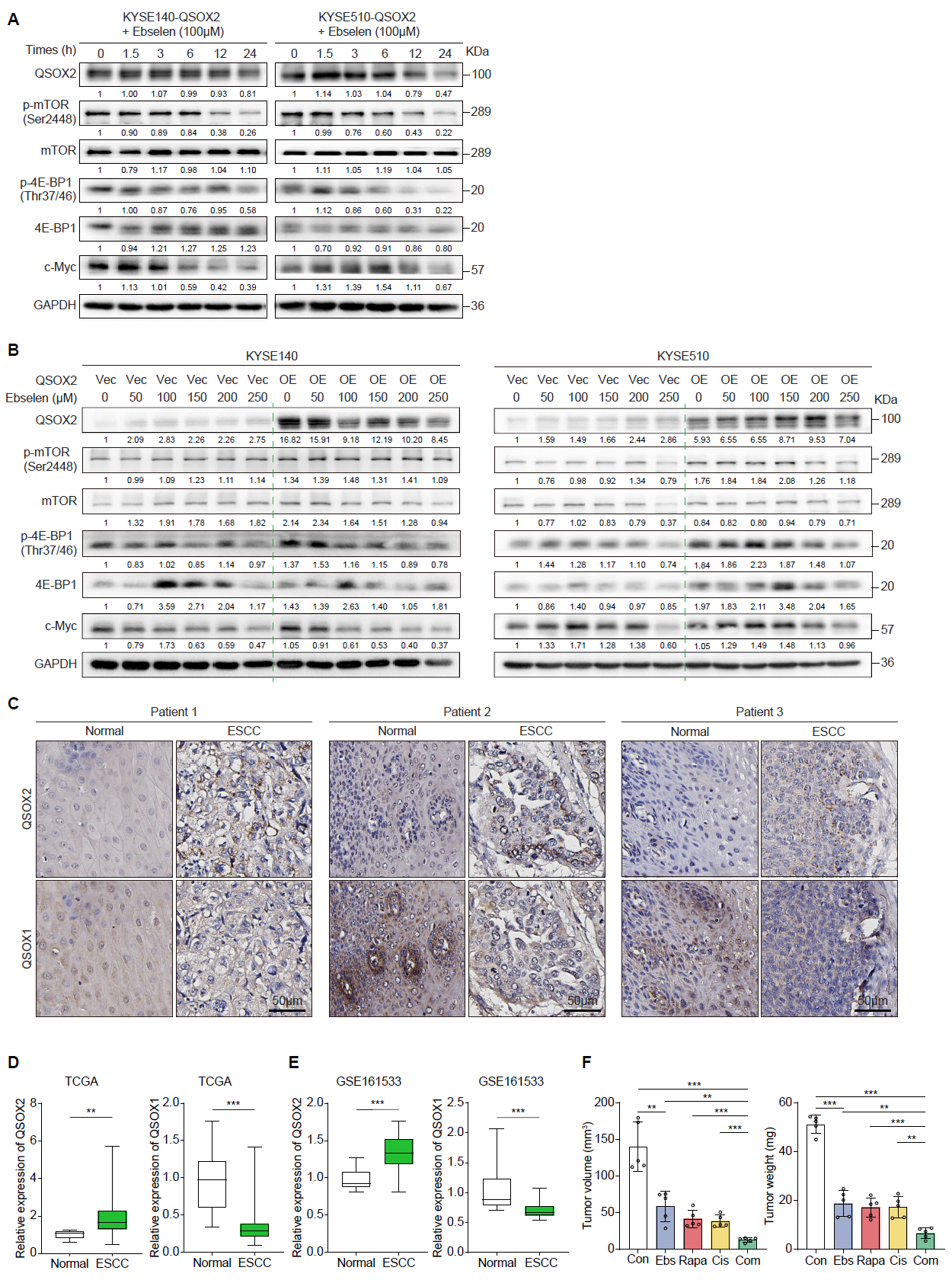


**Fig. S8 Ebselen inhibits QSOX2 activity and ESCC progression.**

**A.** Western blot was performed to test the levels of p-mTOR/p-E-BP1/c-Myc signaling pathway in KYSE140/510-QSOX2 cells treated with different times of Ebselen (24 h).

**B.** Western blot was performed to test the levels of p-mTOR/p-E-BP1/c-Myc signaling pathway in KYSE140/510-Vector and KYSE140/510-QSOX2 cells treated with different concentrations of Ebselen (24 h).

**C.** IHC staining of successive sections revealed the expression of QSOX1 and QSOX2 in ESCC tumors and matched adjacent normal tissues.

**D.** Analysis of TCGA data demonstrated differential expression patterns of QSOX1 and QSOX2 between ESCC tumor tissues and adjacent normal tissues.

**E.** Analysis of GSE161533 data demonstrated differential expression patterns of QSOX1 and QSOX2 between ESCC tumor tissues and matched adjacent normal tissues.

**F.** The tumor volume and weight of KYSE510 xenograft tumor after treatments were calculated. Con, control; Ebs, Ebselen; Rapa, Rapamycin; Cis, Cisplatin; Com, Combination.

In all panels, data are presented as the mean ± SD; In panels **D** and **F**, data were analyzed using unpaired two-tailed Student’s *t*-test with Welch’s correction; In panel **E**, data were analyzed using paired two-tailed Student’s *t*-test with Welch’s correction; ***P* < 0.01, and ****P* < 0.001.

**Table S1**. Antibodies used for western blot

| **Antibodies** | **Corporations** | **Catalog No.** | **Dilution** |
| --- | --- | --- | --- |
| QSOX2 | Abcam | ab121376 | 1:2000 |
| c-Myc | Cell Signaling Technology | 18583 | 1:2000 |
| Sox2 | Cell Signaling Technology | 23064 | 1:2000 |
| CD271 | Beyotime | AG2744 | 1:1000 |
| CD44 | Beyotime | AF0105 | 1:1000 |
| Notch1 | Cell Signaling Technology | 3608 | 1:2000 |
| TSC2 | Cell Signaling Technology | 4308 | 1:2000 |
| p-TSC2 (Ser939) | Cell Signaling Technology | 3615 | 1:1000 |
| p-TSC2 (Thr1462) | Cell Signaling Technology | 3617 | 1:1000 |
| p-TSC2 (Ser1387) | Cell Signaling Technology | 5584 | 1:1000 |
| TSC1 | Cell Signaling Technology | 6935 | 1:2000 |
| mTOR | Cell Signaling Technology | 2983 | 1:2000 |
| p-mTOR (Ser2448) | Cell Signaling Technology | 5536 | 1:2000 |
| p-mTOR (Ser2448) | Abclonal | AP1413 | 1:2000 |
| 4E-BP1 | Cell Signaling Technology | 9644 | 1:2000 |
| p-4E-BP1 (Thr37/46) | Cell Signaling Technology | 2855 | 1:1000 |
| Akt | Proteintech | 60203-2 | 1:2000 |
| p-Akt (Ser473) | Proteintech | 66444-1 | 1:2000 |
| IGF1R | Abcam | ab182408 | 1:1000 |
| p-IGF1R(Tyr1162/1163) | Invitrogen | 12640 | 1:1000 |
| β-Tubulin | Proteintech | 10068-1 | 1:2000 |
| GAPDH | Servicebio | GB12002 | 1:10000 |

**Table S2**. Antibodies used for immunostaining

| **Antibodies** | **Corporations** | **Catalog No.** | **Dilution** |
| --- | --- | --- | --- |
| QSOX2 | Abcam | ab121376 | 1:200 |
| Ki67 | Abcam | ab15580 | 1:400 |
| c-Myc | Abcam | ab32072 | 1:100 |
| Pan-cytokeratin | MXB | RAB-0050 | 1:500 |
| TSC2 | Cell Signaling Technology | 4308 | 1:800 |
| TSC1 | Cell Signaling Technology | 6935 | 1:200 |
| p-TSC2 (Ser939) | Cell Signaling Technology | 3615 | 1:200 |
| p-mTOR (Ser2448) | Abclonal | AP1413 | 1:100 |
| Phospho-p38 MAPK (Thr180/Tyr182) | Proteintech | 28796-1 | 1:100 |
| Phospho-ERK1/2 (Thr202/Tyr204) | Proteintech | 28733-1 | 1:100 |
| Cleaved Caspase-3 | Cell Signaling Technology | 9664 | 1:300 |
| α-SMA | Cell Signaling Technology | 19245 | 1:500 |
| p-IGF1R (Tyr1162/Tyr1163) | Invitrogen | 12640 | 1:100 |

**Table S3**. Univariate and multivariate analyses of overall survival in ESCC patients (n = 95).

| Variable | Univariate analysis | | |  | Multivariate analysis | | | | | |
| --- | --- | --- | --- | --- | --- | --- | --- | --- | --- | --- |
|  | Hazard ratio | 95% CI | *P* value |  | Hazard ratio | | 95% CI | *P* value | |  |
| Gender | 0.190 | 0.044-0.819 | **0.026** |  | 0.276 | 0.062-1.238 | | | 0.093 | |
| Age | 1.036 | 1.000-1.074 | 0.052 |  |  |  | | |  | |
| Weight | 1.003 | 0.975-1.032 | 0.845 |  |  |  | | |  | |
| Smoking history | 0.244 | 0.721-3.604 | 0.244 |  |  |  | | |  | |
| Drinking history | 2.008 | 0.698-5.780 | 0.196 |  |  |  | | |  | |
| TNM stage | 1.749 | 1.089-2.810 | **0.021** |  | 1.382 | 0.819-2.332 | | | 0.226 | |
| Tumor location | 1.043 | 0.556-1.956 | 0.896 |  |  |  | | |  | |
| Histologic grade | 1.124 | 0.768-1.644 | 0.549 |  |  |  | | |  | |
| Lymph node metastasis | 1.781 | 0.887-3.579 | 0.105 |  |  |  | | |  | |
| QSOX2 expression | 2.120 | 1.002-4.486 | **0.045** |  | 1.830 | 0.818-4.091 | | | 0.141 | |

CI, confidence interval; Statistical significance (*P* < 0.05) is shown in bold.

**Table S4.** Summary of mass spectrometry analysis of QSOX2 interactors

| Identified proteins | Unique Petides in KYSE30 | Unique Petides  in KYSE180 | Protein Accession | Protein Description |
| --- | --- | --- | --- | --- |
| **QSOX2** | **16** | **20** | **Q6ZRP7** | **Sulfhydryl oxidase 2** |
| **TSC2** | **12** | **5** | **P49815** | **Tuberin** |
| **TSC1** | **11** | **4** | **Q92574-1** | **Hamartin** |
| XPO1 | 7 | 6 | O14980 | Exportin-1 |
| CNNM4 | 5 | 8 | Q6P4Q7 | Metal transporter CNNM4 |
| CAT | 4 | 5 | P04040 | Catalase |
| GEMIN4 | 4 | 3 | P57678 | Gem-associated protein 4 |
| OSBPL3 | 4 | 3 | Q9H4L5 | Oxysterol-binding protein-related protein 3 |
| SPTAN1 | 4 | 1 | Q13813 | Spectrin alpha chain, non-erythrocytic 1 |
| DDX20 | 3 | 4 | Q9UHI6 | Probable ATP-dependent RNA helicase DDX20 |
| BZW2 | 3 | 2 | Q9Y6E2 | Basic leucine zipper and W2 domain-containing protein 2 |
| UBE2T | 3 | 1 | Q9NPD8 | Ubiquitin-conjugating enzyme E2 T |
| PCBP2 | 2 | 3 | Q15366-3 | Isoform 3 of Poly(rC)-binding protein 2 |
| ABCF3 | 2 | 4 | Q9NUQ8 | ATP-binding cassette sub-family F member 3 |
| CSE1L | 2 | 4 | P55060-1 | Exportin-2 |
| DCUN1D5 | 2 | 4 | Q9BTE7 | DCN1-like protein 5 |
| AHNAK2 | 2 | 2 | Q8IVF2-1 | Protein AHNAK2 |
| PRDX1 | 2 | 2 | Q06830 | peroxiredoxin-1 |
| ARPC4 | 2 | 1 | P59998 | Actin-related protein 2/3 complex subunit 4 |
| XPO5 | 2 | 1 | Q9HAV4 | Exportin-5 |
| UQCRC2 | 2 | 1 | P22695 | Cytochrome b-c1 complex subunit 2, mitochondrial |
| RPL27 | 2 | 1 | P46776 | 60S ribosomal protein L27a |
| RPS20 | 1 | 3 | P60866 | 40S ribosomal protein S20 |
| U2AF1 | 1 | 3 | Q01081 | Splicing factor U2AF 35 kDa subunit |
| PRRC2B | 1 | 1 | Q5JSZ5-1 | Protein PRRC2B |
| RPS13 | 1 | 3 | P62277 | 40S ribosomal protein S13 |
| GEMIN2 | 1 | 2 | O14893 | Gem-associated protein 2 |
| GCDH | 1 | 1 | Q92947-1 | Glutaryl-CoA dehydrogenase, mitochondrial |
| XIRP1 | 1 | 2 | Q702N8-1 | Xin actin-binding repeat-containing protein 1 |
| HIVEP2 | 1 | 2 | P31629 | Transcription factor HIVEP2 |
| RALGAPA2 | 1 | 2 | Q2PPJ7-1 | Ral GTPase-activating protein subunit alpha-2 |
| CDK1 | 1 | 1 | P06493 | Cyclin-dependent kinase 1 |
| RPN2 | 1 | 1 | P04844-1 | Dolichyl-diphosphooligosaccharide--protein glycosyltransferase subunit 2 |
| PDCL | 1 | 1 | Q13371 | Phosducin-like protein |
| KLHL5 | 1 | 1 | Q96PQ7 | Kelch-like protein 5 |
| BRD7 | 1 | 1 | Q9NPI1-1 | Bromodomain-containing protein 7 |
| TBC1D2B | 1 | 1 | Q9UPU7-1 | TBC1 domain family member 2B |
| PLA2G3 | 1 | 1 | Q9NZ20 | Group 3 secretory phospholipase A2 |
| WDFY4 | 1 | 1 | Q6ZS81-1 | WD repeat- and FYVE domain-containing protein 4 |
| ZNF737 | 1 | 1 | O75373 | Zinc finger protein 737 |
| TMEM131L | 1 | 1 | A2VDJ0 | Transmembrane protein 131-like |
| PACSIN3 | 1 | 1 | Q9UKS6 | Protein kinase C and casein kinase substrate in neurons protein 3 |
| SLC17A7 | 1 | 1 | Q9P2U7 | Vesicular glutamate transporter 1 |
| PIAS3 | 1 | 1 | Q9Y6X2 | E3 SUMO-protein ligase PIAS3 |
| UBTF | 1 | 1 | P17480-1 | Nucleolar transcription factor 1 |
| UMPS | 1 | 1 | P11172 | uridine 5'-monophosphate synthase |
| TMED9 | 1 | 1 | Q9BVK6 | Transmembrane emp24 domain-containing protein 9 |
| CCDC171 | 1 | 1 | Q6TFL3 | Coiled-coil domain-containing protein 171 |
| SPEN | 1 | 1 | Q96T58 | Msx2-interacting protein |
| RYR2 | 1 | 1 | Q92736 | ryanodine receptor 2 |
| HOPX | 1 | 1 | Q9BPY8-1 | Homeodomain-only protein |
| TMA16 | 1 | 1 | Q96EY4 | translation machinery-associated protein 16 |
| CTSD | 1 | 1 | P07339 | Cathepsin D |
| DNM1 | 1 | 1 | Q05193-1 | Dynamin-1 |
| RBL2 | 1 | 1 | Q08999 | Retinoblastoma-like protein 2 |
| TTC21B | 1 | 1 | Q7Z4L5 | Tetratricopeptide repeat protein 21B |
| RYR3 | 1 | 1 | Q15413-1 | Ryanodine receptor 3 |
| PALMD | 1 | 1 | Q9NP74-1 | Palmdelphin |
| NRIP1 | 1 | 1 | P48552 | Nuclear receptor-interacting protein 1 |
| MTOR | 1 | 1 | P42345 | Serine/threonine-protein kinase mTOR |
| CNTNAP3 | 1 | 1 | Q9BZ76 | Contactin-associated protein-like 3 |
| TCERG1 | 1 | 1 | O14776-1 | Transcription elongation regulator 1 |
| TFRC | 1 | 1 | P02786 | Transferrin receptor protein 1 |
| CORO1C | 1 | 1 | Q9ULV4-3 | Isoform 3 of Coronin-1C |
| DHCR24 | 1 | 1 | Q15392 | Delta(24)-sterol reductase |
| LRCH3 | 1 | 1 | Q96II8-1 | Leucine-rich repeat and calponin homology domain-containing protein 3 |
| CCDC183 | 1 | 1 | Q5T5S1 | Coiled-coil domain-containing protein 183 |
| EPG5 | 1 | 1 | Q9HCE0-1 | Ectopic P granules protein 5 homolog |
| SCARA5 | 1 | 1 | Q6ZMJ2-1 | Scavenger receptor class A member 5 |
| ZSWIM9 | 1 | 1 | Q86XI8 | Uncharacterized protein ZSWIM9 |
| NEMP1 | 1 | 1 | O14524-1 | Nuclear envelope integral membrane protein 1 |
| MED12L | 1 | 1 | Q86YW9-1 | Mediator of RNA polymerase II transcription subunit 12-like protein |
| SP100 | 1 | 1 | P23497 | Nuclear autoantigen Sp-100 |
| AJUBA | 1 | 1 | Q96IF1 | LIM domain-containing protein ajuba |
| RBMS2 | 1 | 1 | Q15434 | RNA-binding motif, single-stranded-interacting protein 2 |
| SSMEM1 | 1 | 1 | Q8WWF3 | Serine-rich single-pass membrane protein 1 |
| RWDD2A | 1 | 1 | Q9UIY3 | RWD domain-containing protein 2A |
| KMT2D | 1 | 1 | O14686-1 | Histone-lysine N-methyltransferase 2D |
